# Predictions from masked motion with and without obstacles

**DOI:** 10.1371/journal.pone.0239839

**Published:** 2020-11-06

**Authors:** Ariel Goldstein, Ido Rivlin, Alon Goldstein, Yoni Pertzov, Ran R. Hassin

**Affiliations:** 1 Princeton Neuroscience Institute, Princeton University, Princeton, NJ, United States of America; 2 Cognitive Science Department, Hebrew University of Jerusalem, Jerusalem, Israel; 3 Department of Psychology, Hebrew University of Jerusalem, Jerusalem, Israel; 4 James Marshall Chair of Psychology, the Department of Psychology and The Federmann Center for the Study of Rationality, The Hebrew University of Jerusalem, Jerusalem, Israel; Katholieke Universiteit Leuven, BELGIUM

## Abstract

Predicting the future is essential for organisms like *Homo sapiens*, who live in a dynamic and ever-changing world. Previous research has established that conscious stimuli can lead to non-conscious predictions. Here we examine whether masked stimuli can also induce such predictions. We use masked movement–with and without obstacles–to examine predictions from masked stimuli. In six experiments a moving object was masked using continuous flash suppression (CFS). A few hundred milliseconds after the object had disappeared, a conscious probe appeared in a location that was either consistent with the masked stimulus or not. In Experiments 1–3 the movement was linear, and reaction times (RTs) indicated predictions that were based on direction and speed of movement. In Experiment 4, the masked moving object collided with an obstacle and then disappeared. Predictions in this case should reflect deflection, and indeed reaction times revealed predictions on the deflection route. In Experiments 5 and 6 we introduce an innovative way of using eye-tracking during continuous flash suppression (CFS) and report physiological evidence–in the forms of eye-movements–for masked stimuli induced predictions. We thus conclude that humans can use dynamic masked stimuli to generate active predictions about the future, and use these predictions to guide behavior. We also discuss the possible interpretations of these findings in light of the current scientific discussion regarding the relation between masked presentation, subliminal perception and awareness measurement methods.

## Introduction

The ability to predict future events is essential for organisms like the *Homo sapiens*, who live in a dynamic and ever-changing world. We predict the locations of moving objects, allowing us to duck falling bricks and avoid being hit by cars [1]; we predict the next note of a beloved melody [2, 3], the colors and shapes of familiar and unfamiliar scenes [4], and the next word in a sentence [5, 6]. We even automatically predict the goals that propel human action and consequences of our behaviors–predictions that help us navigate the social world [7–9]. These predictions change not only our future behavior, but also our perception [10–14], and it has been suggested that they are a cornerstone of human intelligence [15].

The central role of predictions in our lives has led to the view that generating predictions is the ultimate function of our brains, and hence that investigating the cognitive and brain mechanisms that underlie predictions is a central endeavor of the cognitive and brain sciences [8, 9, 12, 15–20]. Given its centrality, it is not surprising that the different literatures on prediction vary considerably in terms of the questions they ask, the methodologies they use, and even in how they refer to the act of predicting (e.g., anticipating [21], expecting [3], prospecting [22], extrapolating [23, 24] and preparing [25]).

Our focus here is on *active predictions*: predictions that require that one figures out an aspect of the structure of his current environment, and then integrates this understanding with existing knowledge structures to anticipate the future. Intuitively one may hold that although predictions can be nonconscious, humans cannot base predictions on events that they do not fully perceive. To illustrate, batters in baseball might make nonconscious predictions based on a ball’s movement, but (the argument goes) they cannot make these predictions if they do not fully perceive the ball. We challenge this view in six experiments that use continuous flash suppression [26] to mask a moving object. In Experiments 1–3 we examine predictions that are based on direction and speed of masked stimuli. In Experiment 4 we raise the bar, and present a masked moving object that collides with an obstacle. Predictions here must consider a collision-based change of trajectory. In Experiments 5 and 6 we provide evidence from eye-movement supporting the existence of predictions that are based on dynamic masked stimuli. Our experiments suggest that humans can make active predictions that are based on masked stimuli.

We chose to frame our results in terms of predictions that are induced by masked dynamic stimuli and not subliminal stimuli, as there is a vibrant debate regarding the necessary procedures that are needed for establishing subliminality (these issues will be covered at length below, in the Subliminal and Masked Presentation section). Throughout the manuscript we will address the findings that were already framed in the literature as induced by subliminal stimuli as such. We will also describe our efforts to render our stimuli subliminal, and base our conclusion on subliminal trials (as established by various analyses). However, given the ongoing debate, we will frame our general claims using the term masked presentation.

Importantly, the literature of nonconscious processing is not limited to subliminal perception [27–29]. Thus, this literature can motivate both subliminal and masked presentation findings.

### Predictions and conscious awareness

Intuitively the tasks of figuring out the structure of our environment and using it to generate active predictions seem to require full conscious awareness. For example, being conscious of a car’s speed and road’s curve in order to predict the car’s future position. Ample research suggests that this is not the case. It has been repeatedly shown that learning aspects of the structure of our environment can occur without or with reduced awareness of the process, its contents, or of the predictions that have been generated using this information [21, 25, 30–37]

There is one salient exception to this rule: in all previous research on active prediction, the relevant environmental events were supraliminal, that is–clearly conscious. Thus, for example, in scenario-based predictions, the scenarios are always consciously presented [9, 38, 39]. In Reber’s classic grammar-learning studies the stimuli–often letters–were always supraliminal [34, 35], and the same is true for studies that use the serial reaction task in statistical learning [21, 33, 37]. The constant use of supraliminal stimuli for the research of active prediction leaves open the question–is full awareness of the stimulus a prerequisite for generating active prediction.

### Predictions, spatial attention and subliminal stimuli

The last three decades have yielded much evidence regarding our ability to process masked and subliminal stimuli, and the possible effects of these stimuli ([28, 40, 41] but see [42]). The existing data suggest that if a subliminal prime P is associated with (semantic or procedural) knowledge K, than P can activate K [7, 12, 43–47]. To take just a few examples, findings have shown subliminal activation of simple semantic knowledge [40, 48–51], complex stereotypes [52], numbers [51, 53], national ideologies [45, 54], short sentences and arithmetic equations [51], goals [44, 55–57] and even executive functions [47, 58, 59]. It was also shown that humans can learn the association between a subliminal cue and the identity of a correlated following target [60]. It is interesting to note that despite these advances, evidence for active predictions from subliminal stimuli is generally lacking.

It was established that a subliminal stimulus can capture attention and shift spatial attention to the location of the subliminal stimulus. This can facilitate a faster response to a conscious target at the cued location [61–66].

While it was also established that subliminal stimuli can elicit endogenous attention [67], shifting attention to a different location than that of the subliminal stimulus is more complicated. For example, it was demonstrated that if intermixed with congruent conscious symbolic stimuli (e.g. arrow shape), the same type of stimuli can subliminally shift spatial attention toward a possible location of a conscious target that is not at the same position as the subliminal stimuli themselves [68]. However, conflicting evidence has been gathered regarding the ability of masked direction-words (i.e ‘up’, ‘down’) to direct spatial attention [69, 70]. The conclusion from these findings is that while subliminal or masked priming is often being attended (at least spatially), its ability to cause a shift of attention to a different location is not trivial.

### Subliminal and masked presentation

Some of the work examining nonconscious processing relies on subliminal presentation. A discussion regarding whether one can conclude that a group of masked stimuli were rendered subliminal is currently taking place [71–74]. A common approach to using subliminal stimuli to induce nonconscious processing combines two types of measurements. One, is designed to examine whether the masked stimuli were rendered subliminal. These are *awareness* tests, which measure participants’ awareness of masked stimuli. The other is the *effect* measurement, which examines behavioral change (e.g., faster reaction time) induced by masked stimuli [75]. These two measurements are combined to form a conclusion: if the *effect* measurement indicates that stimuli were processed and the *awareness* measurement indicates that stimuli were not consciously perceived (hence subliminal), one can conclude that the stimuli were processed nonconsciously.

There are two types of awareness tests that are commonly used: “subjective” tests and “objective” tests. In subjective awareness tests, after each masked trial participants are asked to report if or to what degree they perceived the masked stimulus [62, 63, 76–78]. In “objective” awareness tests, one takes inability to discriminate, rather than subjective reports, as evidence for subliminality. The measurement is being done in a separate part of the experiment (than the one that examines the influence of the masked stimulus on behavior). For example, one infers (un)awareness from the performance in a forced-alternative-choice paradigm that tests the detection of some aspect of the masked stimulus [41, 51, 79–81].

The proper and exhaustive ways of measuring awareness are still researched and the questions regarding measures and analyses is still open [72, 82]. While this question is still unresolved, we will mention that for experiments that used masked presentation and reaction time as the dependent variable, objective measures of awareness were more commonly used than the subjective ones [40, 51, 81, 83, 84].

When using an objective test, one should adopt a chance level threshold. Participants who score appreciably above chance level according to the objective measurement are considered to be conscious of the stimuli and the researcher removes their data from the critical analysis. Then, only participants who are classified as not conscious of the stimuli are included in the final analysis from which evidence for nonconscious processing is drawn. While this methodological strategy is highly prevalent [40, 41, 45, 80, 81, 83–85], it is not clear how this threshold should be chosen [74, 86–89].

When one uses a threshold method as part of the objective test, there is always a risk of classifying participants as nonconscious despite them being aware of a small number of trials (in the experimental block). Theoretically, this may lead to the erroneous conclusion that their observed behavior stems from nonconscious processing when in fact it stems from the small number of trials they were aware of. This concern is directly outlined by Shanks [74]. Shanks claims that the group of nonconscious participants is chosen based on an extreme mean value. Hence, due to regression to the mean, the group’s true awareness value is less extreme. The true and less extreme value may be above chance level and is not indicative of subliminality.

In light of this criticism, guaranteeing we included only completely subliminal stimuli is beyond the reach of the paradigms we use here. Yet, our paradigms, measurements and the analyses we conduct below, provide support for the idea that the phenomenon we are examining is non-conscious. We offer several types of analyses to support the claim that the measured behavior is indeed a result of nonconscious processing.

First, the main analysis compares the performance of participants whose behavior on an awareness test indicated they were not aware of the masked stimuli (classified as nonconscious). Second, acknowledging the risk of including trials for which the mask did not render subliminal presentation (i.e. supraliminal or conscious), we offer a novel simulation-based analysis that examines the likelihood of getting the measured effect due to a number of conscious trials, assuming that subliminal trials do not contribute to a systematic effect. Third, we examine the behavioral effects for participants who were not classified as nonconscious (i.e. classified as conscious). If one believes that the effects that are measured for the group that was classified as nonconscious is due to a number of conscious trials, one would expect a (larger) effect for a group of participants who were aware of the masked stimuli. Fourth, we implement a regression based analysis [90], that avoids the use of choosing a threshold and infers the existence of a nonconscious effect from a regression between the behavioral effect and awareness score in the objective test. We also discuss the limitations of this method and our suggestion to control for them. Lastly, we perform aggregated analyses that allow us to examine the effects for a big sample of participants, as well as adopting conservative criteria for unawareness of the stimuli. Together, we believe the analyses support evidence for the claim that the effects measured are due to nonconscious processing. However, as we cannot state with full certainty that all trials were rendered subliminal we will address the evidence as evidence for prediction induced by masked motion.

### The present paper

In the present paper, we argue that humans can generate active predictions from masked motion. The logic that led us to our prediction is simple, though. Human consciousness is severely limited in terms of its available resources for high-level cognitive processes (e.g., we can hold only one verbal thought at a time; [91–93]. Leaving active predictions to the domain of conscious processes therefore seems unwarranted. This basic argument has recently led one of us [28, 94] to suggest that nonconscious processes can carry out every fundamental, basic-level function that conscious processes can carry out (but see [42]) This perspective, predicts that humans can generate active predictions even from events they do not consciously experience.

To test these ideas, we examined movement-based predictions. Predictions induced by moving objects guide human motor behavior, perception [13, 95] and the understanding of complex human behavior [96, 97]. An ample body of evidence documents human’s perception and memory tendency to displace the final position of a target in the direction of a previously moving probe [98–100]. It was suggested that this tendency enables the cognitive system to generate near-future predictions [101]. Importantly, this vast literature covers evidence for predictions that require internalization of momentum law [100] including the case of bouncing of a barrier [102].

With one exception we discuss below, the literature on movement-based predictions used supraliminal stimuli to show that humans spontaneously and effortlessly generate active predictions. While the stimuli are always supraliminal, there seems to be an agreement that the predictions themselves are not necessarily conscious. That is, when we move our hand to grasp a moving ball, the computations that underlie our predictions are not consciously available to us.

Motion happens in space and time, and hence creating subliminal motion requires keeping the stimulus below the threshold of consciousness for relatively long durations. Historically, subliminal presentations could only be very short, on the order of tens of milliseconds. It was therefore almost impossible to study non-conscious movement (but see [103]). The development of Continuous Flash Suppression (CFS; [26]) changed this state of affairs. In CFS each eye is presented with its own input. One eye is presented with the stimuli one wishes to render subliminal (i.e., the prime). The other eye is presented with an attention-grabbing, constantly changing set of stimuli that mask the prime. This methodology creates masking that may last up to a few seconds [26, 51, 104, 105].

Cognitive scientists quickly realized the advantages of CFS and used it to study non-conscious movement. Using various methodologies (including CFS and binocular rivalry), it has been shown that we can non-consciously perceive motion [106–110]. There is only one paper we are aware of that examined active predictions from subliminal motion. The results led the authors to conclude that we *cannot* generate such predictions [62]. We return to this paper in the General Discussion.

In six experiments we show evidence gathered from behavioral and physiological measures for predictions that are based on speed and direction of masked movement. In all experiments, a CFS-masked object moves on a (virtual) line, and then disappears. Participants are then asked to perform a simple discrimination in a location that is either predicted by the movement, or not. Moreover, the fourth experiment takes masked stimuli-based predictions one step further by masking a moving probe that collides with a masked obstacle and then disappears. In this case, predictions must take deflection into account. The following experiments demonstrate masked stimuli based predictions using eye-tracking and provide converging evidence for masked stimuli based prediction. Together, these experiments provide strong support for the idea that active predictions can be based on masked movement.

## Experiment 1

Experiment 1 examines whether we can extract the direction of a masked moving object, and whether we can predict its future location on this route. Participants were presented with CFS-masked moving probes. 250 milliseconds after the movement ended, a supraliminal target appeared on the screen. Targets could appear in one of three locations: (i) in a consistent “future” location on the route of the movement (*future* condition); (ii) in another equally distant location, perpendicular to the probe’s route (*control* condition); and (iii) in a “past” equally distant location that the probe had already passed through (*past* condition). Our main prediction had to do with facilitation in a future location relative to the control. Such a facilitation would show that participants predicted the future location of the moving object. Following the same logic, and the vast literature on inhibition of return [111–113] and Representational Momentum effect [114, 115], we also predicted that participants would react faster to targets in *future* locations relative to *past* ones.

### Method

#### Participants

Thirty students (fifteen males; M = 23.31 years, SD = 2.43) with intact vision (without glasses or contact lenses) participated in the experiment. They received either 10 NIS (~$3) or course credit. Given that we had no prior experiment of this sort, we predetermined the number of participants to thirty students. Prior to their participation all participants gave an informed consent by signing a written consent form.

#### Ethics statement

All Experiments were ethically approved by the ethics committee of the psychology department of the Hebrew University headed by Prof. Jonathan Huppert.

#### Apparatus

Experiments 1–4 stimuli were presented on a 15-inch CRT monitor (refresh Rate = 100Hz; resolution = 1024 X 768) controlled by a Psychtoolbox extension for MATLAB [116]. The monitor was fitted with a mirror stereoscope (at a distance of 30 cm) to allow stimuli to be presented monocularly.

#### Procedure

**Fig 1:**
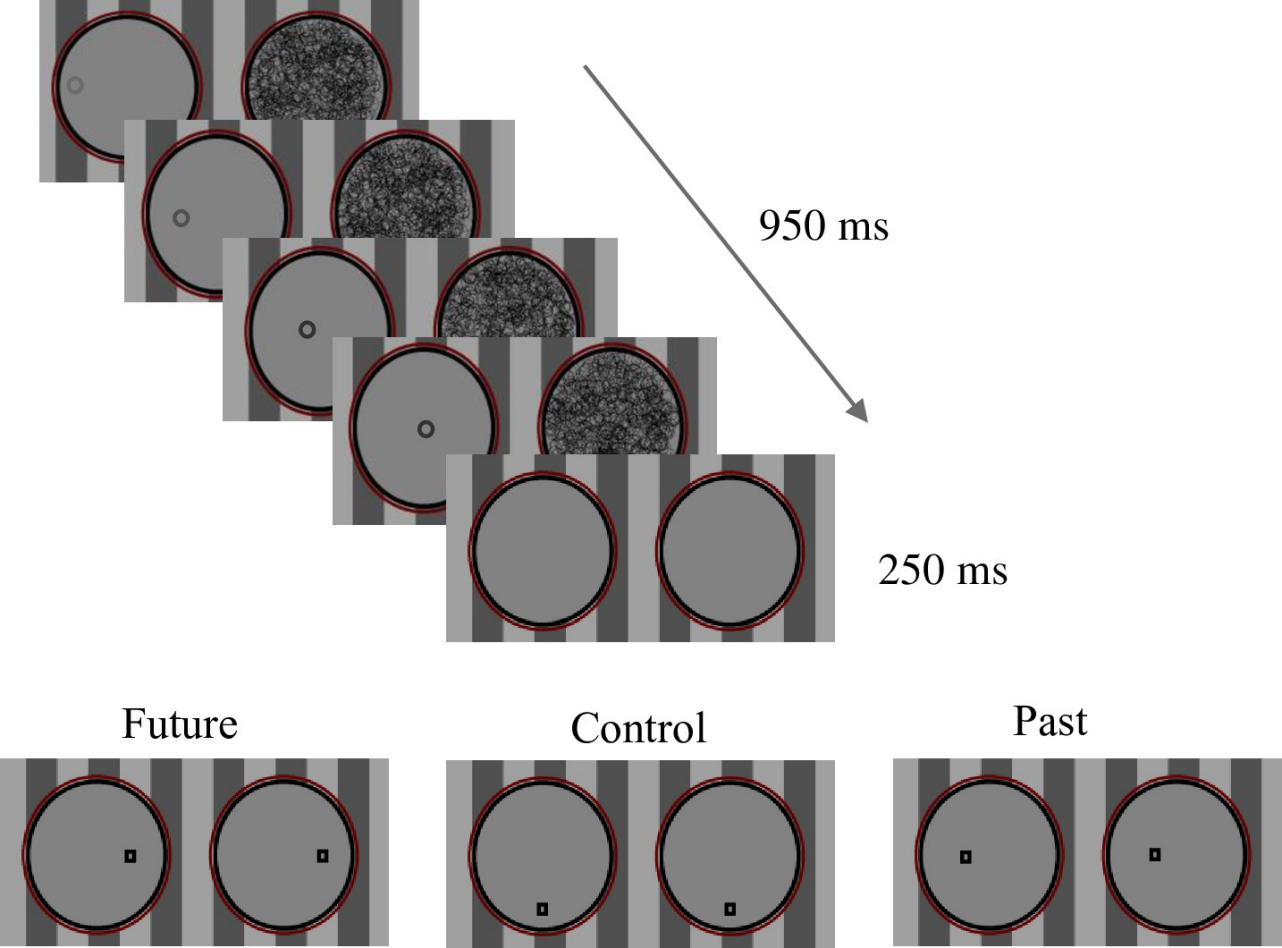
Description of the different conditions. The prime is masked and it moves on a straight route. The target is presented to both eyes; thus, it is consciously perceived. In the *past* condition the target appeared in a location that the masked probe had moved through. In the *future* condition the target appeared in an expected location on the route. Finally, in the *control* condition the target appeared in a perpendicular location to the route. All locations were equidistant from the last location of the prime.

The experiment consisted of 3 phases (see Fig 1):

*Phase 1*: *Supraliminal training*. This phase consisted of 64 trials. On each trial a probe (a circle with a radius of five pixels, 0.25°) moved on the screen starting at a distance of 95 pixels (4.79°) from the fixation point and ending at fixation (velocity of 5.04° per second). Eight different starting locations were equally spaced around the fixation point in different orientations (0°, 45°, 90°, 135°, 180°, 225°, 270°, 315°). The probe moved in a straight line for 950 milliseconds. Its contrast gradually increased, starting from the background color which was set to 50% contrast (making it completely invisible as it was the same color of the background) and going up to 78% contrast (the higher the contrast the darker the probe is). The probe then disappeared. After 250 milliseconds a target (either a square or a diamond) appeared in one of four possible equidistant locations, 50 pixels (2.52°) from the fixation. In the *future* condition, the target appeared in a location that was consistent with the probe’s direction of movement (50% of the trials). In the *past* condition the target appeared in a location along the probe’s route (25% of the trials). Lastly, in the *control* condition the target appeared in a location that was perpendicular to the probe’s route (25%). Since there are two possible perpendicular points for each route, the probe appeared 12.5% of the time in each. Note, that like in the classic Posner cost-benefit paradigm [117], the distribution of location is unequal, in order to encourage using the movement as a cue. Participants were asked to perform a classification task as fast as they could, and indicate whether the target was a rectangle or a diamond (each appeared 50% of the time). Reaction times (RTs) and accuracy were recorded.

*Phase 2*: *Masked presentation*. This phase consisted of 128 trials. The timing and presentation of the stimuli were identical to those of the conscious phase, except that primes were masked using continuous flash suppression. Reaction times and errors rates were recorded. The data collected in this phase are the data we used to test our main hypotheses.

*Phase 3*: *Awareness test*. We used an “objective test” to measure awareness. The objective test was a two-interval forced-choice judgment test [118, 119] and it consisted of 64 trials. Each trial consisted of two intervals (randomly ordered): an *empty interval* and a *full interval*. The full intervals were identical to the trials in the masked phase with one exception: there was no supraliminal target. In empty intervals the masking was identical, but there was no moving probe. Participants were asked to indicate whether the prime appeared in the first interval or in the second. We compared each participant’s accuracy to that expected by chance using the binomial distribution. Participants whose accuracy did not deviate from chance (accuracy level of less than 60%) were classified as nonconscious [51, 120].

We also used subjective measures at the end of the experiment. Given the nature of our design, including trial-by-trial subjective measures [62, 63, 121] did not seem feasible. First and foremost, because we did not want to draw participants’ attention to the primes. And secondly, because adding these measures might affect RTs. Indeed, the vast majority of experiments that use trial-by-trial subjective measures do not examine RTs.

### Results & discussion

#### Data preparation

One participant was excluded due to faulty recording. Nine participants whose scores in the awareness test (phase 3) deviated from chance were classified as conscious, and the rest were classified as nonconscious. We emphasize that being classified as nonconscious does not suggest absolute certainty that all trials were subliminal as explained in the introduction, and that a small portion of trials may render themselves as visible. Two participants whose accuracies were below 90% in the masked presentation (phase 2) were excluded from analyses [51]. These cleaning procedures left us with eighteen participants for the main analyses.

Erroneous trials (i.e., trials in which participants made mistakes in the classification task (2.21%) and trials with reaction times (RTs) longer than 5 seconds or shorter than 0.2 seconds (0.04%) were excluded from analyses. Then, trials with RTs that deviated more than 3 standard deviations from each participant’s mean were excluded (1.3%). For identical cleaning procedures see Sklar et al. [51].

#### Awareness

The nonconscious classified group’s mean accuracy in the awareness test (M = .48, SD = 0.06) did not deviate from chance (t(17) = 1.52, p = .15).

#### Main results

**Fig 2:**
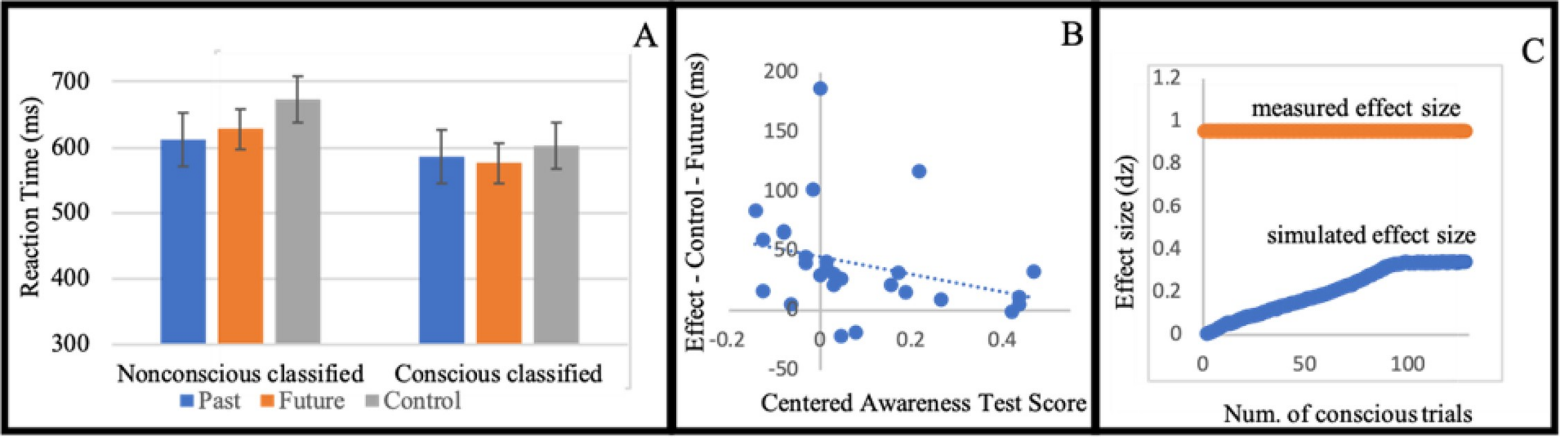
(A) Mean reaction time for condition (standard error to each side). (B) Each dot represents a participant. The horizontal axis (x-axis) represents the centered Awareness score. The vertical axis is the behavioral effect of the participant. The intercept is the predicted effect for a participant who is unaware to the stimuli. The value of 0.1 on the horizontal axis is the frequentist threshold. (C) The horizontal value (x-axis) represents the number of simulated conscious trials (by sampling from the supraliminal training phase). The vertical axis is the measured effect size. In blue we mark the average effect size for 10,000 iteration for each sample size. The standard error is smaller than 0.01 for each simulated number of conscious trials. In red is the measured effect size of the actual masked part.

Supporting our hypothesis, participants who were classified as nonconscious were faster in the future condition (M = 0.63 seconds, SD = 0.18) than in the control condition (M = 0.67 seconds, SD = 0.19) (t(17) = 4.05, p < .001, d = 0.95). There was no effect on accuracy (t<1). This result indicates that participants predicted the future location of the probe, and used this prediction to improve their performance (repeated measure analysis with Angle factor for Experiments 1, 2, 3, 5, 6 presented in Tables 1–5 in S2 Appendix respectively). As expected, the past condition (M = 0.61 seconds, SD = 0.16) also yielded faster responses than the control condition (t(17) = 4.72, p < .001, d = 1.11). There was no effect on accuracy (t<1). Unexpectedly, however, participants were faster in the past condition than in the future condition (t(17) = 2.47, p = .024, d = 0.58), see Fig 2A. This result does not challenge our main hypothesis regarding non-conscious active prediction. Re-examining the literature, we found contradicting evidence regarding the past locations. On the one hand, the literature on inhibition of return suggests inhibition of responses to past location [111–113]. On the other hand, this result can be viewed as a replication of the finding that awareness is not necessary for directing spatial attention produced by Hsieh and Colas [62]. In addition, the attentional momentum effect literature, which mainly demonstrates future prediction, has also provided evidence for facilitation of past locations [13, 99]. This is an ongoing debate [122, 123] that is beyond the scope of this manuscript. Given that the past condition is not central to our hypothesis, we do not use it in the following experiments.

#### Further analyses

It was recently claimed that the commonly used procedure of excluding participants according to their performance on an awareness test phase leads to errors in inferring nonconscious effect [74]. In order to deal with such criticism we implemented an analysis inspired by Greenwald et al.’s suggestion [90]. This analysis includes all participants and does not require exclusion of participants based on the performance on an awareness test phase. It involves regressing the predicted effect (RT control–RT future) on a centered awareness test score (awareness test phase performance minus 0.5). Note that this regression also includes the nine participants who performed better than chance on the awareness test. Greenwald et al. (1995) suggest viewing the intercept as a measure of processing without awareness: if it significantly deviates from zero, then there is an effect even on the theoretical point of no awareness (chance performance in the awareness test). The intercept was in fact significant, (b0 = 45.60, t(25) = 4.99, p < .001). The slope did not turn out significant (b = -74.63, t(25) = 1.63, p = .116). For scatter plot see Fig 2B.

It has been argued that the regression analyses developed by Greenwald overestimate the intercept [124–126]. This criticism depends on two necessary conditions. First, they require a positive correlation between the effect and the awareness test score; otherwise, conscious trials cannot drive the effect and the intercept is not overestimated. Second, there should be an error measurement in the measures of awareness.

The first condition does not hold in our data. Namely, the correlation between the effect and awareness test score is negative as is evident by the negative slope at Fig 2B, albeit not significantly (r(25) = -.309, p = .116). However, we were able to determine that there is strong evidence that the correlation is in fact not positive using Bayes factor (The calculation of Bayes factor was done using a free online available JASP). The Bayes factor of the hypothesis that the correlation is not positive is 9.89 (Bayes factor above 3 is considered as strong evidence). Given these two analyses, it is clear that the correlation is not positive, and hence Greenwald analysis is valid for this experiment. Furthermore, while there is no agreed upon method to correct the error measurement, it is worth mentioning that the reliability of the awareness test is 0.895 (Chronbach alpha). This strongly suggests that the second condition does not apply to our data as well.

The influence of increased level of awareness as reflected in the awareness score can be examined directly by replicating the analysis on the participants who were classified as conscious. Participants in this group were marginally significantly faster in the future condition (M = 0.58 seconds, SD = 0.078) than in the control condition (M = 0.6 seconds, SD = 0.1) (t(8) = 2.26, p = .054, d = 0.75). Past condition (M = 0.59 seconds, SD = 0.1) did not yield significantly faster responses than the control condition (t(8) = 1.26, p = .244, d = 0.42). Past and future conditions did not yield a significant difference (t<1). Due to the fact that these participants were marginally significantly faster in the future condition in comparison to the control condition, we turned to compare this effect between the conscious and nonconscious classified participants.

Given that the past condition is not central to our hypothesis we conducted a mixed design with the participants’ awareness classification (nonconscious classified, conscious classified) as a between-subject condition and experimental condition (control, future) as a within-subject condition.

The logic behind these analyses is simple. If one holds that the effects we report are driven by conscious participants, then the effects should be stronger for those participants (compared to non-conscious participants). As in both awareness conditions the comparison between control condition and future condition turned significant, the within-subject factor turned significant as well (F(1,25) = 16.12, p < .001, partial eta sq. = 0.392). The between-subject factor did not turn significant (F(1,25) = 1.04, p = .315, partial eta sq. = 0.034). Interestingly, the interaction did not turn significant as well (F<1). As it is not possible to draw conclusions of a lack of interaction and given that the participants were not randomly assigned to the two awareness levels the lack of interaction should be interpreted cautiously. However, if one believes that consciousness drives this effect, one may expect to see bigger difference between control and future condition in the conscious classified condition than the nonconscious classified.

Another concern that may arise in masked experiments is that a few conscious trials (trials where the mask did not render the stimuli subliminal) drive the measured effect in the masked phase. We offer here a novel simulation-based analysis training phase and simulate the effect measured by a few conscious trials that are mixed with non-conscious trials that do not contribute to a systematic effect. The supraliminal training phase consists of 64 trials and it shares the same visual attributes of the masked stimuli in the masked phase. In essence it is the same experiment as the masked experiment but without masking (and fewer trials).

In this simulation we estimated the effect size as computed by Cohen’s d for standardized difference score (because the analysis is within participant) for different samples of conscious trials. We manipulate the number of conscious trials that are mixed in the masked phase and compute the effect size. Let’s describe the simulation for the hypothesis that participants are aware of one trial. First, we shuffle the labels of the conditions *future* and *control* for all the trials in the masked part (this is shuffled data). This disconnects the relation between the reaction times recorded and labels of the conditions and is in line with the null hypothesis. Then we randomly chose one trial from the supraliminal training. From this trial we took the reaction time and condition and replaced one trial of the same condition in the shuffled data. This simulates the situation where subliminal trials (trials in which the mask rendered the stimuli subliminal) do not contribute to a systematic effect (shuffling the labels). Then we perform the same preprocess procedure and analysis (of effect size). We repeat this process 10,000 times for each possible number of trials (1 to 128). In Fig 2C we present the expected effect size of different number of conscious trials (in blue) and the actual effect size measured (in red). No number of simulated conscious trials yields the effect size measured in the masked phase. While this may seem surprising, in the supraliminal training phase the same participants were only marginally significant faster in the future condition (M = 0.57 seconds, SD = 0.11) than in the control condition (M = 0.59 seconds, SD = 0.12) (t(18) = 1.78, p = .092, d = 0.42).

To sum up, the analyses support our main hypothesis. Participants predicted a future location of a masked moving probe and successfully used it to improve performance. We described several analyses that reduce the chance of the effect to be driven by conscious trials thus we believe that one can take these findings as evidence for nonconscious prediction induced by dynamic subliminal stimuli. However, we acknowledge that there is a small chance that some trials were in fact supraliminal.

## Experiment 2

One alternative explanation for the results of Experiment 1 holds that they do not reflect predictions, but rather a preference for the line on which the probe moved. This is the case, one might argue, because 75% of the targets (those of the *future* and *past* conditions) appeared on this virtual line. Note that in order to use this bias one would have to maintain at least two points on the route of the movement in the order of their appearance (otherwise one would not know where the “future” location was). Yet, in order to rule out this alternative interpretation empirically, Experiment 2 had only two conditions–future and control–that were equally likely. In the future condition the target appeared in a location that was consistent with the probe’s direction of movement (50% of the trials). In the control condition the target appeared in an equidistant location that was inconsistent with the route (perpendicular to it; 50%). Unlike Experiment 1, we used only one of the two possible perpendicular locations, and hence the likelihood of the target appearing on a future location or on a control one was identical.

### Method

#### Participants

Since Experiment 2 is a replication of Experiment 1, we used the same predetermined number of participants. Thirty students (fourteen males; M = 24.82 years, SD = 3.54) with intact vision (without glasses or contact lenses) participated in the experiment. They received either 10 NIS (~$3) or course credit. Prior to their participation all participants gave an informed consent by signing a written consent form.

#### Procedure

Experiment 2 is identical to Experiment 1 except for the following changes. First, the targets were equally distributed between the *future* and the *control* conditions. Second, we changed the supraliminal training (phase 1), such that the movement was not on the same lines as that in the masked presentation (phase 2). The directions of the masked presentation in Experiment 2 were the same as Experiment 1.

### Results & discussion

#### Data preparation

Exclusion procedures were identical to those of Experiment 1. Two participants did not comply with the instructions of the experiment and a third had faulty recording and so their data were removed from the analyses. Sixteen participants whose awareness test score deviated from chance were classified as conscious, and the rest were classified as nonconscious. These cleaning procedures left us with eleven participants in the main analyses. Errors (1.64%), trials with RTs longer than 5 seconds or shorter than 0.2 second (0%), and trials with RTs that deviated more than 3 standard deviations (1.64%) from the participant’s mean were excluded.

#### Awareness

The nonconscious classified group’s mean accuracy in the awareness test (M = .52, SD = 0.05) did not deviate from chance (t(10) = 1.22 p = .25).

#### Main results

**Fig 3:**
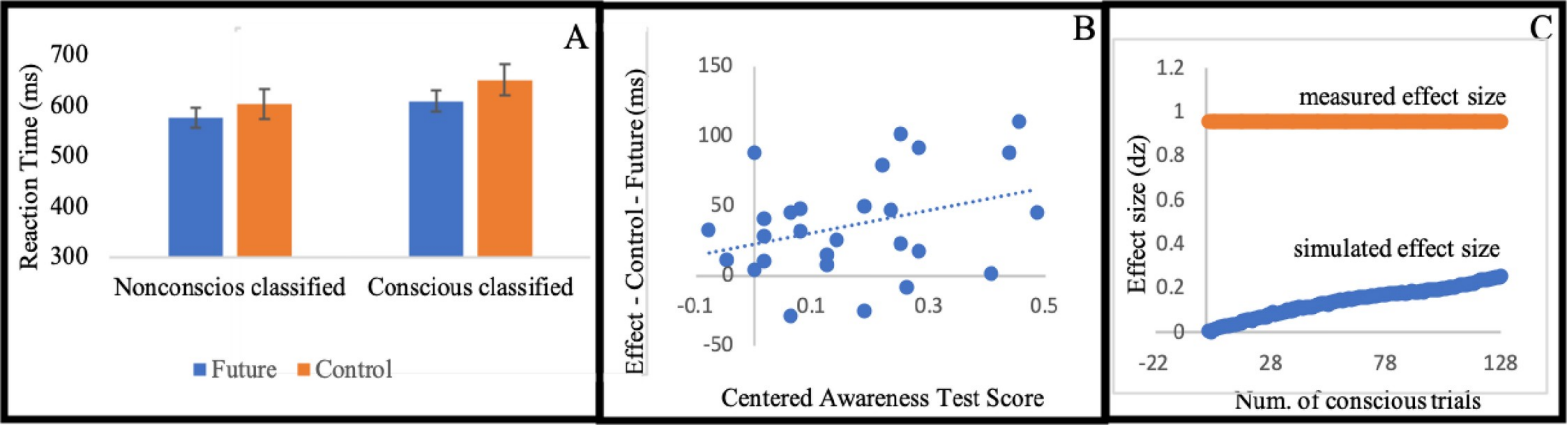
(A) Mean reaction time for condition (standard error to each side). (B) Each dot represents a participant. The horizontal axis (x-axis) represents the centered Awareness score. The vertical axis is the behavioral effect of the participant. The intercept is the predicted effect for a participant who is unaware of the stimuli. The value of 0.1 on the horizontal axis is the frequentist threshold. (C) The horizontal value (x-axis) represents the number of simulated conscious trials (by sampling from the supraliminal training phase). The vertical axis is the measured effect size. In blue we mark the average effect size for 10,000 iteration for each sample size. The standard error is smaller than 0.01 for each simulated number of conscious trials. In red is the measured effect size of the actual masked part.

Supporting our hypothesis, participants who were classified as nonconscious were faster in the *future* condition (M = 0.57 seconds, SD = 0.08) than in the *control* condition (M = 0.60 seconds, SD = 0.06), (t(10) = 3.17, p = .01, d = 0.96), see Fig 3A. No effect on accuracy was found (t <1). These results replicate those of the first experiment, and rule out the alternative interpretation suggested above, according to which the result of Experiment 1 stemmed from induced preference to the line on which the movement occurred.

#### Further analyses

As in Experiment 1, we used Greenwald et al.’s [90] regression analysis to examine the effect (note that this regression includes the sixteen participants who performed better than chance on the awareness test). This regression revealed a significant intercept (b0 = 23.24, t(25) = 2.28, p = .031; see Experiment 1). The slope turned marginally significant (b = 79.83, t(25) = 1.78, p = 0.086). These results serve as further indication that the effect we report here is non-conscious in nature. For scatter plot see Fig 3B.

In this experiment the correlation between the effect and awareness test score is positive and not far away from significance (r(25) = .34, p = .086). However, The Bayes factor of the hypothesis that the correlation is not positive is 0.54 and the Bayes factor that the correlation is positive is 1.84. In Bayesian terms it seems that there is not enough data to determine whether Shanks’ criticisms apply here. Second, the reliability of the awareness test is 0.874 (considered high for psychological measures).

The influence of increased level of awareness as it is reflected in the awareness score can be examined directly by replicating the analysis on the participants who were classified as conscious. The participants in this group were significantly faster in the future condition (M = 0.61 seconds, SD = 0.1) than in the control condition (M = 0.65 seconds, SD = 0.12) (t(15) = 4.047, p = .001, d = 0.87). This motivates a comparison of this effect between the participants who were classified as conscious and nonconscious.

We conducted a mixed design with the participants awareness classification (nonconscious classified, conscious classified) as a between-subject condition and experimental condition (control, future) as a within-subject condition. As in both awareness conditions the comparison between control condition and future condition turned significant, the within-subject factor turned significant as well (F(1,25) = 23.3, p < .001, partial eta sq. = 0.482). The between-subject factor did not turn significant (F(1,25) = 1.137, p = .36, partial eta sq. = 0.043). Interestingly the interaction did not turn significant as well (F<1). While it is not possible to draw conclusions from not getting a significant result, if one believes that consciousness drives this effect, one may expect to see an interaction between level of awareness and the difference between reaction time to future and control conditions. Thus, it does not appear that awareness of the stimuli is the major reason driving the effect for the group of participants classified as nonconscious. However, this analysis should be interpreted cautiously as participants were not randomly assigned to the two awareness levels.

Following the same simulation procedures described in Experiment 1, we examine the possible influence of conscious trials in the masked phase by simulating the effect size that would have been measured if conscious trials were mixed with subliminal trials (that do not contribute to a systematic effect). The simulation suggests that conscious trials are not likely to account for the effect measured in the masked phase (Fig 3C). In the supraliminal training phase participants where not significantly faster in the future condition (M = 0.55 seconds, SD = 0.07) than in the control condition (M = 0.56 seconds, SD = 0.07) (t(10) = 0.96, p = .36, d = 0.29). Which explains why the simulation suggested that conscious trials are not driving the effect measured in the masked phase.

## Experiment 3

Experiments 1 and 2 examined predictions that are based on the direction of movement. The literature in representational momentum documented speed-based predictions in addition to direction-based ones [14, 127, 128]. In Experiment 3 we examine the use of speed of movement in generating active predictions. The masked probes moved on a straight route, following the same spatial patterns of Experiments 1 and 2. However, in contrast to Experiments 1 and 2, half of the probes moved fast, and the other half moved more slowly. The conscious target appeared in a location that was always consistent with the route. Yet, it was either consistent with the speed or inconsistent with it. We hypothesized that performance in consistent trials would be better than that in inconsistent ones.

### Method

#### Participants

Given that the number of participants being excluded from the main analysis increased in Experiment 2 in comparison to Experiment 1 we increased the sample size of Experiment 3. Fifty students (sixteen males; M = 24.23 years, SD = 3.34) with intact vision (without glasses or contact lenses) participated in the experiment. They received either 15 NIS (~$4) or course credit. Prior to their participation all participants gave an informed consent by signing a written consent form.

#### Procedure

As in Experiments 1 and 2, the probe moved on a straight line, starting its movement from a point around the fixation, was masked for 950 milliseconds, and ended its movement at fixation. The fast probe moved at a speed of 3.78° per second, and the slow probe moved at 1.26° per second. 250 milliseconds after the movement ended, a supraliminal target appeared. The target appeared in a location that was either consistent with the probe’s speed or inconsistent with it. Consistent trials were either slow probes followed by targets close to fixation (0.76°) or fast probes followed by targets further away from fixation (2.37°). The masked presentation (phase 2) consisted of 128 trials. 75% of the targets were consistent with the probe’s speed and 25% were inconsistent. Importantly, as half of the probes were fast, and half were slow, the same number of targets appeared in both locations. Thus, even though location and speed are confounded they are not biasing any specific location and do not provide a confounding explanation that avoids the extraction of the speed of the masked stimuli.

### Results & discussion

#### Data preparation

Exclusion procedures were identical to those in Experiments 1 and 2. One participant didn’t comply with the instructions (he took his head off the stereoscope), and two participants had faulty recording, and so their data were removed from the analyses. No participant had accuracy level below 90%. Nineteen participants whose awareness test score deviated from chance were classified as conscious, and the rest were classified as nonconscious. These cleaning procedures left us with twenty-eight participants for the main analyses. Errors (3.43%) and trials with RTs longer than 5 seconds or shorter than 0.2 second (0.22%) were excluded from the analyses. Then, trials with RTs that deviated more than 3 standard deviations from each participant’s mean were excluded (1.9%).

#### Awareness

The nonconscious classified group’s mean accuracy in the awareness test (M = .51 seconds, SD = 0.05) did not significantly deviate from chance, (t(27) = 1.37, p = .18).

#### Main results

**Fig 4:**
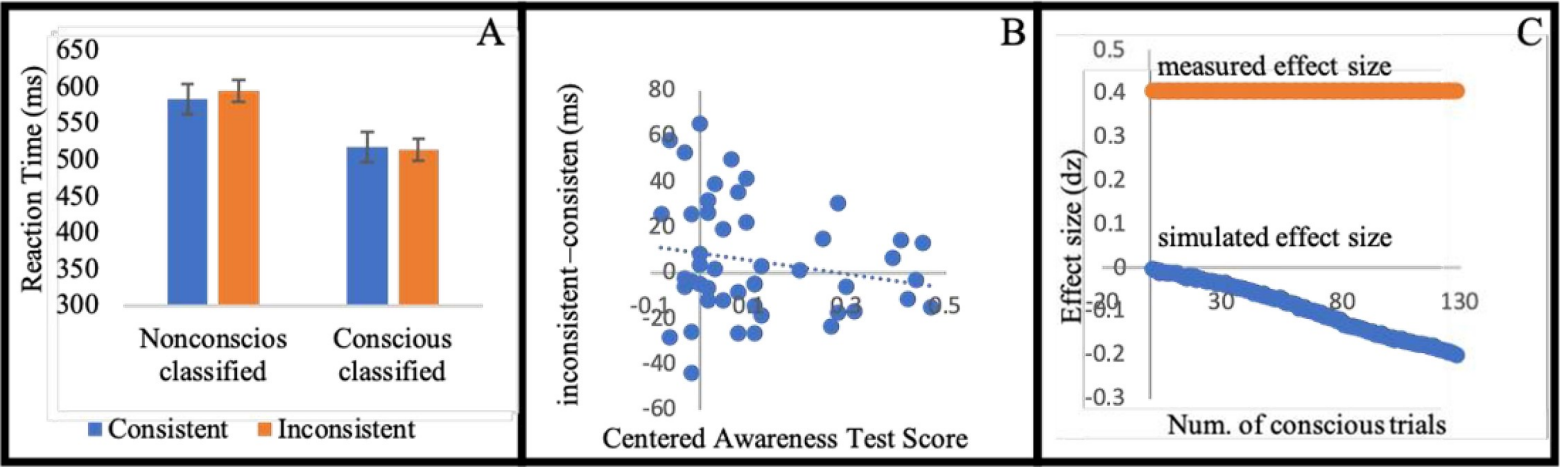
(A) Mean reaction time for condition (standard error to each side). (B) Each dot represents a participant. The horizontal axis (x-axis) represents the centered Awareness score. The vertical axis is the behavioral effect of the participant. The intercept is the predicted effect for a participant who is unaware of the stimuli. The value of 0.1 on the horizontal axis is the frequentist threshold. (C) The horizontal value (x-axis) represents the number of simulated conscious trials (by sampling from the supraliminal training phase). The vertical axis is the measured effect size. In blue we mark the average effect size for 10,000 iteration for each sample size. The standard error is smaller than 0.01 for each simulated number of conscious trials. In red is the measured effect size of the actual masked part.

Supporting our hypothesis, participants who were classified as nonconscious were faster in the consistent (M = 0.58 seconds, SD = 0.1) than in the inconsistent condition (M = 0.59 seconds, SD = 0.11) (t(27) = 2.15, p = .041, d = 0.4). No effect on accuracy was found (t<1). These results, then, suggest that active predictions can be based on speed of movement, and not only on direction of movement. See Fig 4A.

#### Further analyses

As in the previous experiments, we conducted the regression analysis inspired by Greenwald et al. [90], including the nineteen participants who did better than chance on the awareness test, and the intercept was marginally significant (b0 = 8.79, t(45) = 1.917, p < .064). The slope did not turn out significant (b = -30.31, t(45) = 1.29, p = .2). See Fig 4B.

Next, we turn to testing the necessary conditions for Shank’s criticism and the criticism regarding the overestimation of the intercept. First, in this experiment the correlation between the effect and awareness test score is negative but not significant (r(45) = -.19, p = .262). However, The Bayes factor of the hypothesis that the correlation is not positive is 11.78 (Bayes factor above 3 is considered as strong evidence). This is enough to reject both criticisms. Second, the reliability of the awareness test (alpha Cronbach) is 0.864 (considered high for psychological measures).

The influence of increased level of awareness as it is reflected in the awareness score can be examined directly by replicating the analysis on the participants who were classified as conscious. The participants in this group were not significantly faster in the consistent condition (M = 0.52 seconds, SD = 0.1) than in the inconsistent condition (M = 0.51 seconds, SD = 0.11). In fact, they demonstrated the opposite pattern, however, it was not significant (t(18) = 1.2, p = .246, d = 0.26). While not getting a significant effect, is not evidence for the lack of the effect, it weakens the claim the conscious trials are the driving factor of this effect.

We conducted a mixed design with the participants awareness classification (nonconscious, conscious) as a between-subject condition and experimental condition (consistent, inconsistent) as a within-subject condition. The within-subject factor did not turn significant (F(1,45) = 1.078, p = .305, partial eta sq. = 0.023). The between-subject factor turned significant (F(1,45) = 7.55, p = .033, partial eta sq. = 0.144), suggesting that participants classified as conscious were faster than participants classified as nonconscious. The interaction turned significant as well (F(1,45) = 7.55, p = 0.009, partial eta sq. = 0.097). If one believes that conscious trials are responsible for the effect, one would expect to get a significant effect in the conscious classified condition. However, effect was not found for participants classified as conscious. In addition, the interaction was found significant, suggesting that for participants classified as nonconscious, there is a higher difference between inconsistent and consistent conditions, compared with participants classified as conscious. This finding weakens the claim that the main effect is stemming from several conscious trials in the data of participants classified as nonconscious. As mentioned before, this analysis should be interpreted cautiously as participants were not randomly assigned to the two awareness levels.

As in previous experiments, we control for the possible influence of conscious trials in the masked phase by simulating the effect size that would have been measured if conscious trials were mixed with subliminal trials (that do not contribute to a systematic effect). The simulation suggests that conscious trials are not likely to account for the effect measured in the masked phase (Fig 4C). In the supraliminal phase participants where not significantly faster in the future condition (M = 0.56 seconds, SD = 0.1) than in the control condition (M = 0.55 seconds, SD = 0.1) (t(27) = 1.45, p = .156, d = -0.28). In fact, the average indicated the opposite direction (as also reflected in Fig 4C). As noted, it is problematic to rely on significant interaction between the awareness levels and experimental conditions because participants are not randomly assigned to the two levels of awareness. However, it hints that the difference between the consistent and inconsistent conditions is different for high and low awareness conditions. In addition, the difference between consistent and inconsistent conditions did not turn significant for conscious-classified. Thus, it is possible that the non-significant opposite-effect obtained in the conscious-classified emanates from statistical noise. This non-significant opposite effect will explain why the simulated effect size is diverting from the actual effect size.

## Experiment 4

**Fig 5:**
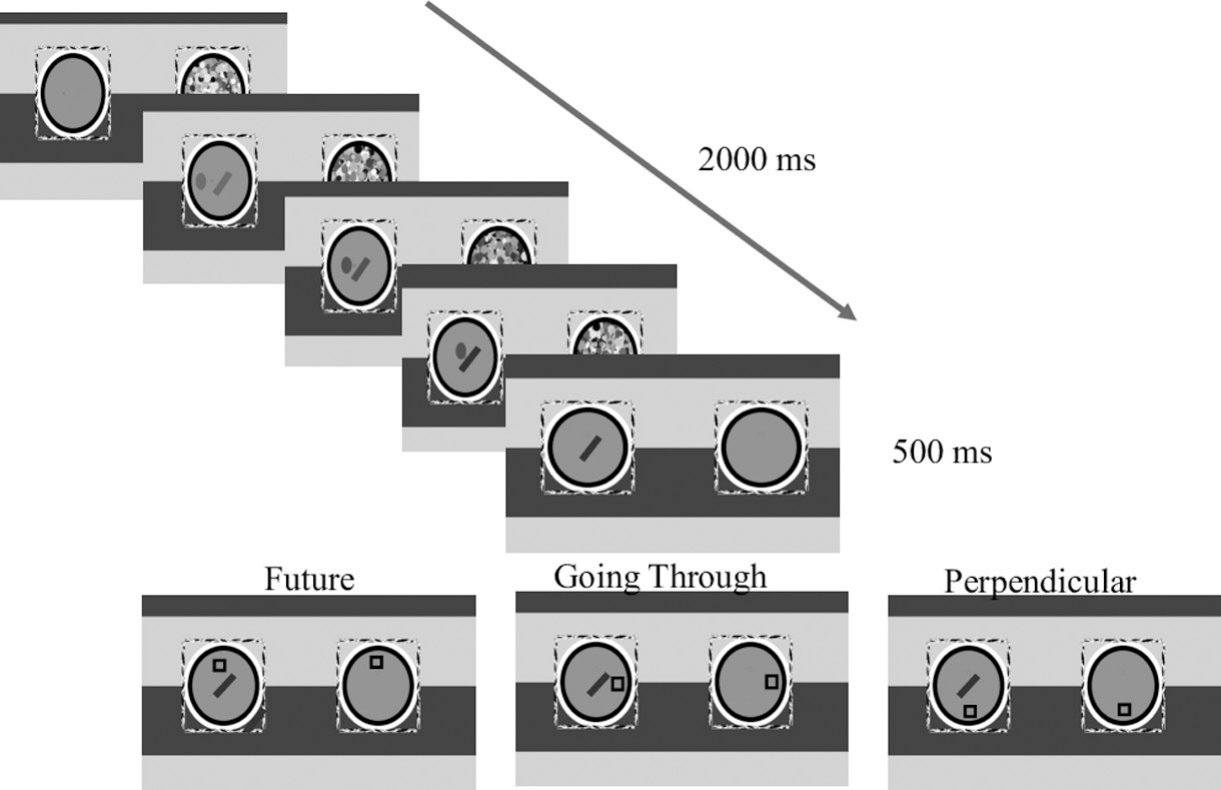
Description of the different conditions. The prime is masked and it moves on a straight route. In the *future* condition the target appears perpendicular to the ball’s motion in the location consistent with bouncing from the obstacle. In the *Perpendicular* condition the target appears perpendicular to the linear motion (but on the other side of the future condition). In the *Going through* condition the target appears in continuation of the linear motion, as if it had gone through the obstacle.

Experiment 4 significantly raises the bar in our examination of active predictions induced by masked motion stimuli. In all previous experiments the object moved on a line, and the supraliminal target appeared on that line. In Experiment 4 we presented a masked moving ball that then hit a masked obstacle (and then disappeared; see Fig 5). The prediction in this case should take bouncing into account: It should not be on the line of the movement but rather on the path created by the bounce. To generate such predictions, one needs to extract and use not only the direction and speed of movement (as examined in Experiments 1–3), but also the angle at which the object hits the obstacle. Furthermore, correct predictions require modulation of the motion’s direction and speed and using a deflection model.

### Method

#### Participants

Given the prolonged masking interval we increased the sample size. Sixty students (thirty-five males; M = 23.52 years, SD = 1.87) with intact vision (without glasses or contact lenses) participated in the experiment. They received either 15 NIS (~$4) or course credit. Prior to their participation all participants gave an informed consent by signing a written consent form.

#### Procedure

*Training*. Experiment 4 shared the motion paths of Experiment 1. Because we introduced an obstacle condition, we made minor changes to the supraliminal training phase. These included: (i) 12 trials in which participants saw a moving “ball” that then collided with an obstacle and bounced from it. 500 milliseconds after the collision the ball disappeared and participants were asked to perform a simple classification task in this location. Because we added this stage, we shortened the supraliminal training (the one we used in the previous experiments) to 16 trials. In these 16 trials the ball disappeared after colliding with the obstacle, and the conscious target appeared in a location that was either consistent or inconsistent with the movement. Again, participants were asked to perform the same discrimination task of indicating whether the conscious target is square shaped of rhombus shaped.

Given the need for modulation of the linear predictions that were introduced in Experiment 1 and Experiment 2 : we increased motion duration to 2 seconds, the collision lasted 200 milliseconds and was followed by 500 milliseconds delay between the end of the collision and the appearance of the target (the ball does not start the deflection motion when masked). Because of this increase in the experiment’s duration we decreased the number of trials to 96. Lastly, the mask consisted of full circles in different shades, and the probe was a full circle too. See Fig 5.

In the test blocks, each trial began with masked linear motion (see Experiments 1 and 2). An obstacle–a bar oriented 45° relative to the probe’s route–was masked as well. In this configuration, a moving ball would have bounced perpendicularly to its original direction of movement. This experiment had two conditions. In the *future* condition the target appeared in a location that was consistent with bouncing (66.66% of the trials). We had two types of control trials. In the first type, *Perpendicular condition*, (16.66% of the trials), the target appeared in a location that was perpendicular to the probe’s route, but in the inconsistent location. In the second type, *Going Through condition*, the target appeared in a location that was consistent with the probe’s direction of movement–as if it went through the obstacle (16.66%). As the directions were fully counterbalanced, the targets were equally likely to appear in all of the possible locations. We used the two conditions because we wanted to explore potential differences between them: Although both locations are inconsistent with the movement itself, the latter might be more accessible because it is on the route of movement. Our main hypothesis is that participants would be faster in the future condition in comparison to the control trials. To clarify, there are four possible obstacles: “-“, “\”, “|” and “/”. However, there are eight possible directions of movement. Each obstacle can be coupled with two possible moving probes. For example, the “\”-obstacle may appear with motion “->”-motion direction or”<-“-motion-direction. So, in fact the obstacle orientation does not cue a specific location.

### Results & discussion

#### Data preparation

Exclusion criteria were identical to those in Experiments 1–3. Excluded from analyses were one participant who took his head out of the binoculars and one participant whose accuracy was below 90%. Thirty-one participants whose awareness test score deviated from chance were classified as conscious, and the rest were classified as nonconscious. One participant had lower than 90% accuracy and higher than chance performance. Errors (2.4%) and trials with RTs longer than 5 seconds or shorter than 0.2 second were excluded from the analyses (less than 0.1%). Then, trials with RTs that deviated more than 3 standard deviations from each participant’s mean were excluded (1.8%).

#### Awareness

The nonconscious classified group’s mean accuracy in the awareness test (M = .52, SD = 0.06) did not significantly deviate from chance, (t(27) = 1.90, p = .07).

#### Main results

**Fig 6:**
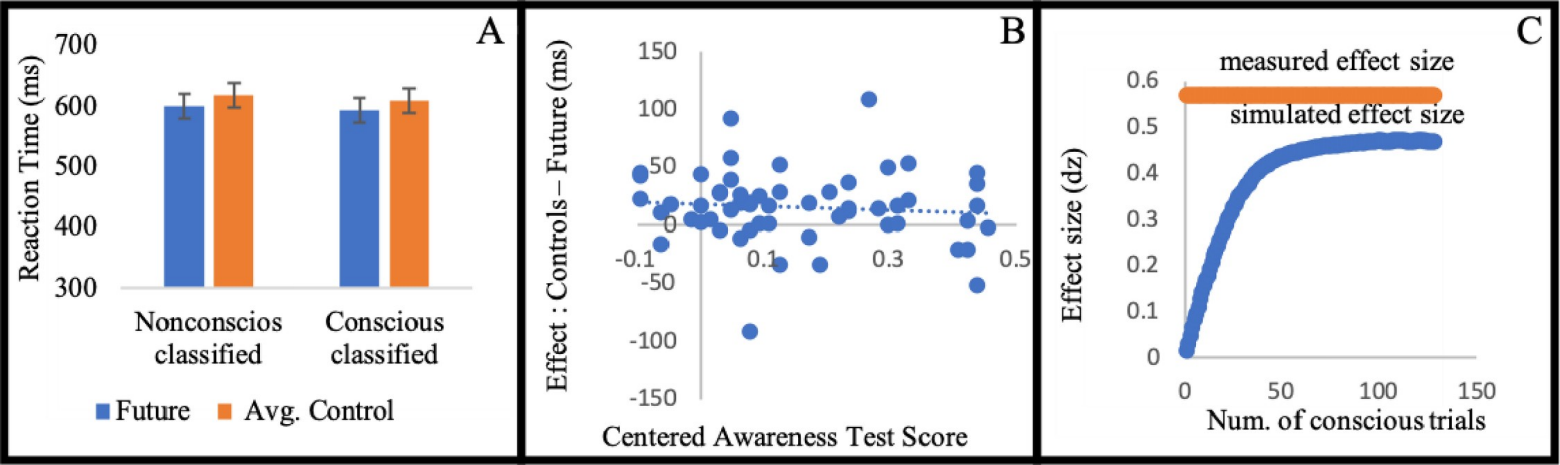
(A) Mean reaction time for condition (standard error to each side). (B) Each dot represents a participant. The horizontal axis (x-axis) represents the centered Awareness score. The vertical axis is the behavioral effect of the participant. The intercept is the predicted effect for a participant who is unaware of the stimuli. The value of 0.1 on the horizontal axis is the frequentist threshold. (C) The horizontal value (x-axis) represents the number of simulated conscious trials (by sampling from the supraliminal training phase). The vertical axis is the measured effect size. In blue we mark the average effect size for 10,000 iteration for each sample size. The standard error is smaller than 0.01 for each simulated number of conscious trials. In red is the measured effect size of the actual masked part.

As there were no significant differences between the two control conditions (t<1) we combined them. Supporting our hypothesis, a within-subject contrast showed that participants who were classified as nonconscious were faster in the future condition (M = 0.6 seconds, SD = 0.1) in comparison the average of the two types of control conditions (Type 1 control trials M = 0.62 seconds, SD = 0.12, and Type 2 M = 0.61 seconds, SD = 0.12), (t(27) = 2.90, p = .007, d = 0.57). See Fig 6A.

#### Further analyses

As in previous experiments—we also conducted the regression analysis [90]. As in previous analyses the independent variable was the awareness level in the awareness test. The dependent variable was the subtraction of the mean RTs in the future condition from the mean RTs in the control conditions. This analysis revealed a significant intercept (b0 = 18.50, t(55) = 3.195, p = .002). The slope did not turn significant (b = -18.37, t(55) = 0.7, p = 0.49). See Fig 6B.

We turn to testing the necessary conditions for Shank’s criticisms and the criticism regarding the overestimation of the intercept. First, in this experiment the correlation between the effect and awareness test score is negative but not significant (r(55) = -.09, p = .49). However, The Bayes factor of the hypothesis that the correlation is not positive is 10.58 (Bayes factor above 3 is considered as strong evidence). This is enough to reject both criticisms. Second, the reliability of the awareness test is 0.874 (considered high for psychological measures).

The influence of increased level of awareness as it is reflected in the awareness score can be examined directly by replicating the analysis on the participants who were classified as conscious. The participants in this group were significantly faster in the future condition (M = 0.59 seconds, SD = 0.09) than in the control condition (M = 0.6 seconds, SD = 0.1) (t(28) = 2.43, p = .022, d = 0.46). Due to the fact that the participants that were classified as conscious showed a similar pattern as the participants that were classified as nonconscious we turned to compare the effect between the two groups. If one believes that the effect obtained for the participants that were classified as nonconscious is due to conscious trials, one would expect to obtain a larger effect in the group that was classified as conscious.

We conducted a mixed design with the participants awareness classification (nonconscious, conscious) as a between-subject condition and experimental condition (average control, future) as a within-subject condition. As in both awareness conditions the comparison between control condition and future condition turned significant, the within-subject factor turned significant as well (F(1,55) = 14.16, p < .001, partial eta sq. = 0.205). The between-subject factor did not turn significant (F<1). Interestingly the interaction did not turn significant either (F<1). While it is not possible to make conclusions of a lack of interaction, if one believes that consciousness drives this effect, one may expect to see an interaction between level of awareness and the difference between reaction time in future and control conditions. However, this analysis should be interpreted cautiously as participants were not randomly assigned to the two awareness levels.

We control for the possible influence of conscious trials in the masked phase by simulating the effect size that would have been measured if conscious trials were mixed with subliminal trials (that do not contribute to a systematic effect). As in previous experiments the simulation suggests that conscious trials cannot account for the effect measured in the masked phase (Fig 6C). In the supraliminal training phase participants were also significantly faster in the future condition (M = 0.59 seconds, SD = 0.1) than in the control condition (M = 0.68 seconds, SD = 0.18) (t(27) = 2.56, p = .017, d = 0.49). However, the simulation suggests that the size effect measured in the masked phase is not likely to account for the effect measured in the masked phase.

## Experiment 5

The main objective of Experiments 5 and 6 is to examine active predictions using physiological measures that will not only corroborate the behavioral results but may also be more sensitive. Eye-tracking has been used in the past to demonstrate our brain’s ability to predict future positions of moving objects, as well as the role of prediction in human behavior [14, 100, 129–132]. It has been demonstrated that eye movements can uncover non-conscious processing, including information that is masked by CFS [133–136] and backward masking [137]. Thus, we used the methodology used in Experiments 1 and 2, this time tracking participants’ eye movements.

### Method

#### Participants

Given the new technology implemented we increased the sample size in comparison to Experiments 1 and 2, as this experiment follows the same design. Forty students (seventeen males; M = 26.92 years, SD = 2.23) with intact vision (without glasses or contact lenses) participated in the experiment. They received either 10 NIS (~$3) or course credit. Prior to their participation all participants gave an informed consent by signing a written consent form.

#### Apparatus

**Fig 7:**
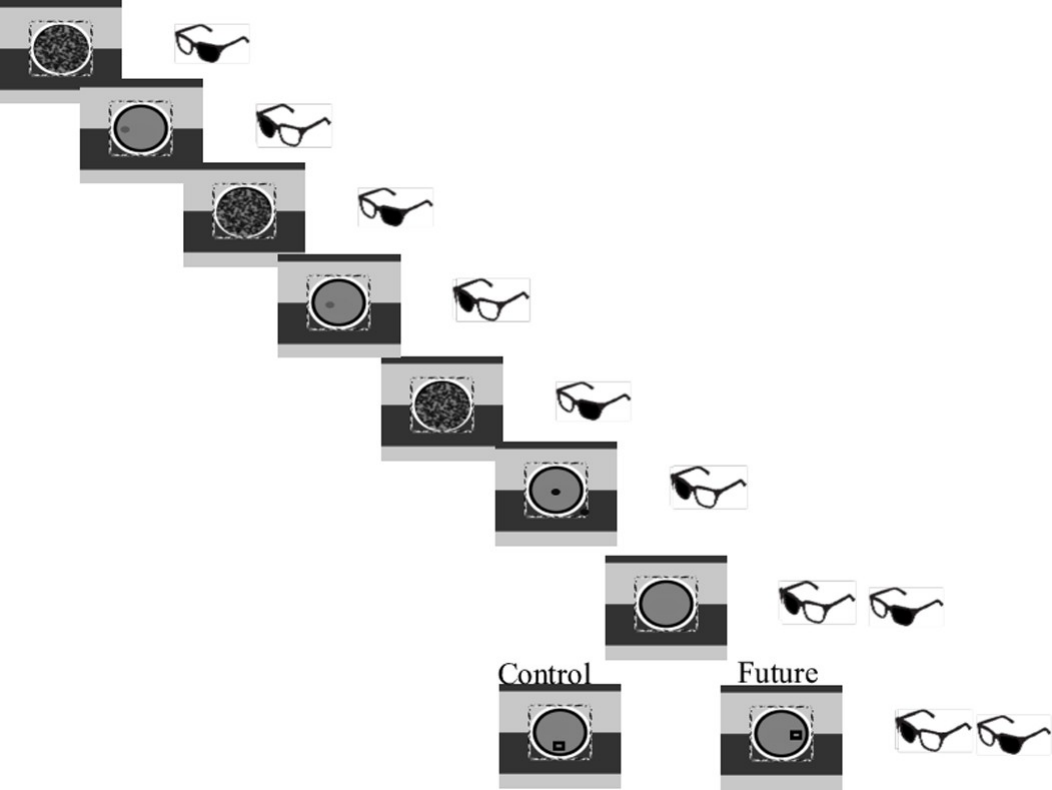
Description of the different conditions. The prime is masked and it moves on a straight route. The target is presented to both eyes; thus, it is consciously perceived. In the *future* condition the target appeared in an expected location on the route. In the *control* condition the target appeared in a perpendicular location to the route. All locations were equidistant from the last location of the prime.

In Experiments 5 and 6 stimuli were presented in 3D monitor (viewing distance = 60 cm) and infra-red emitter (distance = 50 cm). In addition, shutter glasses that communicated with the monitor were used. The monitor was a flat-screen 51cm X 29 cm 3D monitor with a refresh rate of 120 Hz. 1-pixel equal 0.024-degree in the visual field. The monitor alternated between presenting the moving probe and presenting the mask. The shutter glasses’ lenses lighten and darken in 60 Hz in synchrony with the monitor, such that one eye is presented with the mask and the other with the moving probe (see Fig 7). Eye movement recordings were recorded from the eye presented with the probe, using an Eyelink 1000 infrared system (SR Research, Ontario, Canada), with a sampling rate of 1000 Hz and a spatial resolution of less than 0.01°. A standard nine-point calibration was performed at the beginning of the experiment. Stimulus presentation was controlled by psychophysics toolbox extension for MATLAB [116].

#### Procedure

The experimental conditions of this experiment were identical to those of Experiment 2. 75% of the targets were in the *future* condition and 25% in the *control* condition. Due to the technical effects of shadowing in the 3d monitor (the alternation of the screen was not perfect. Some “residue” of the image presented in one frame appeared together with the alternate image presented in the next frame), we needed to change the visual features of the probe and mask, both of which were filled (and not empty as in Experiments 1–3). The period of time between the disappearance of the masked probe and appearance of the conscious target was 260 milliseconds. See Fig 7.

### Results & discussion

#### Data preparation

Tracking the eyes while wearing shuttering glasses introduced some technical challenges. Eight participants were removed from the analysis as the eye-tracker lost tracking during the experiment (this was later solved by monitoring the eye-tracking during the experiment for each participant). Exclusion procedures were identical to those of Experiments 1–4. Twelve participants whose scores in the awareness test (phase 3) deviated from chance were classified as conscious, and the rest were classified as nonconscious. Two participants whose accuracy was below 90% in the masked presentation (phase 2) were excluded from the analyses too. After the exclusion of participants, erroneous trials (i.e., trials in which participants made mistakes in the classification task) were excluded from the analyses (2.5%), as were trials with reaction times (RTs) longer than 5 seconds or shorter than 0.2 seconds (less than 0.5%), and likewise trials with RTs that deviated more than 3 standard deviations from each participant’s mean (1.8%).

#### Preprocess of eye-tracking data

We conducted three pre-processing procedures. First, we eliminated epochs with blinks. Blinks were defined as intervals of 50 ms or less in which the gaze exited the screen.

**Fig 8:**
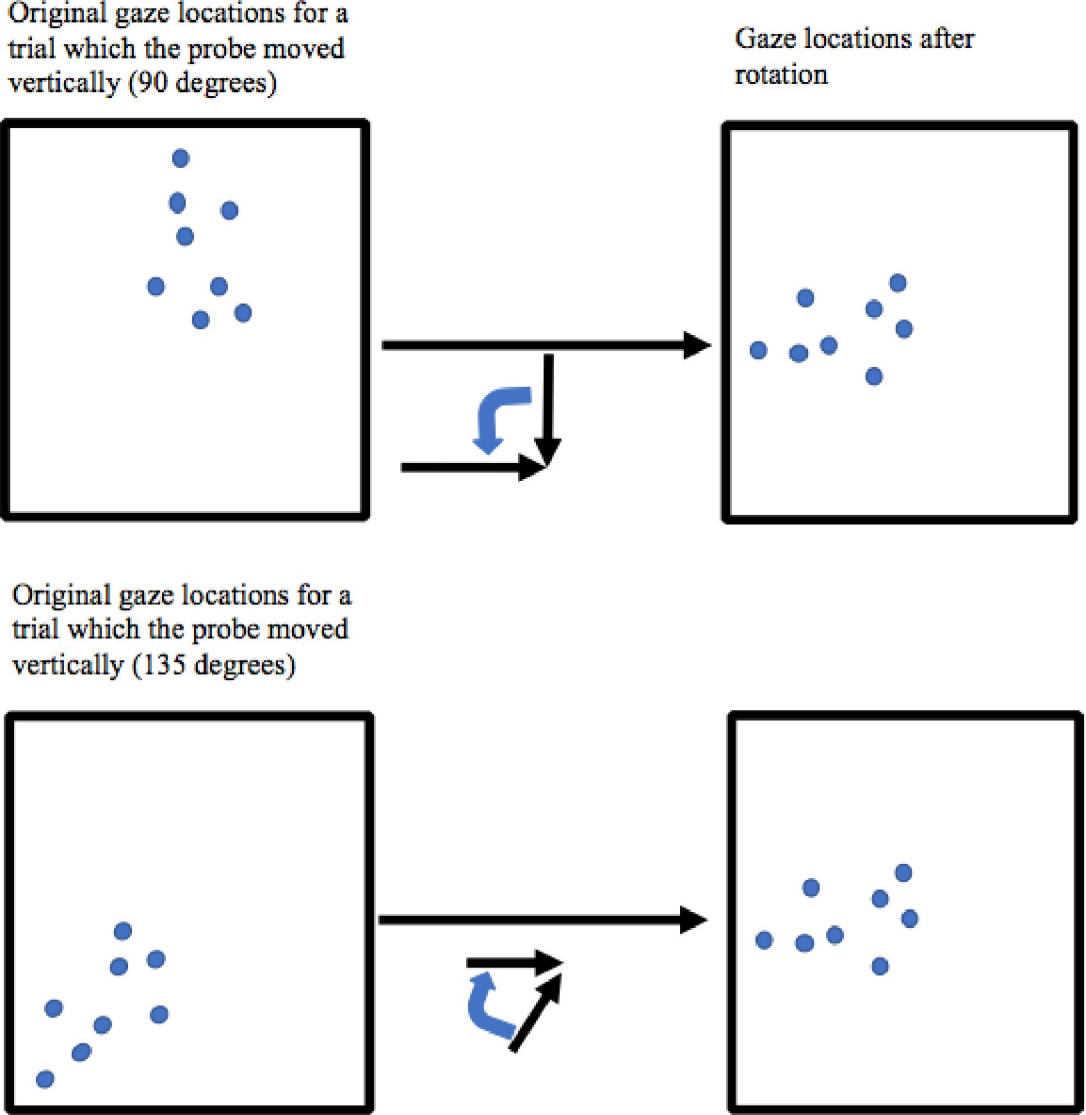
Two examples of data rotation. Original data on the left, and rotated data on the right.

Second, for ease of analysis and interpretation, we performed a rotation of the eye-tracking data. The motion of the probes was in one of eight different directions, the rotations aligned all movements as if from left to right. For example, recordings from trials in which the probe moved from the top of the screen to the center (forming a 90-degree angle with the horizontal axis) were rotated 90 degrees to the left around the fixation point which is “origin” (see Fig 8). Third, because we wanted to be cautious about possible asynchronies between the eye-tracking and experimental computer, we eliminated the first and last 25 data points of the movement and of the interval between movement and target (the pattern of results remains similar with no such precautions).

#### Awareness

The nonconscious classified group’s mean accuracy in the awareness test (M = .50, SD = 0.04) did not deviate from chance (t(17) = 0.33, p = .74).

#### Main results

**Fig 9:**
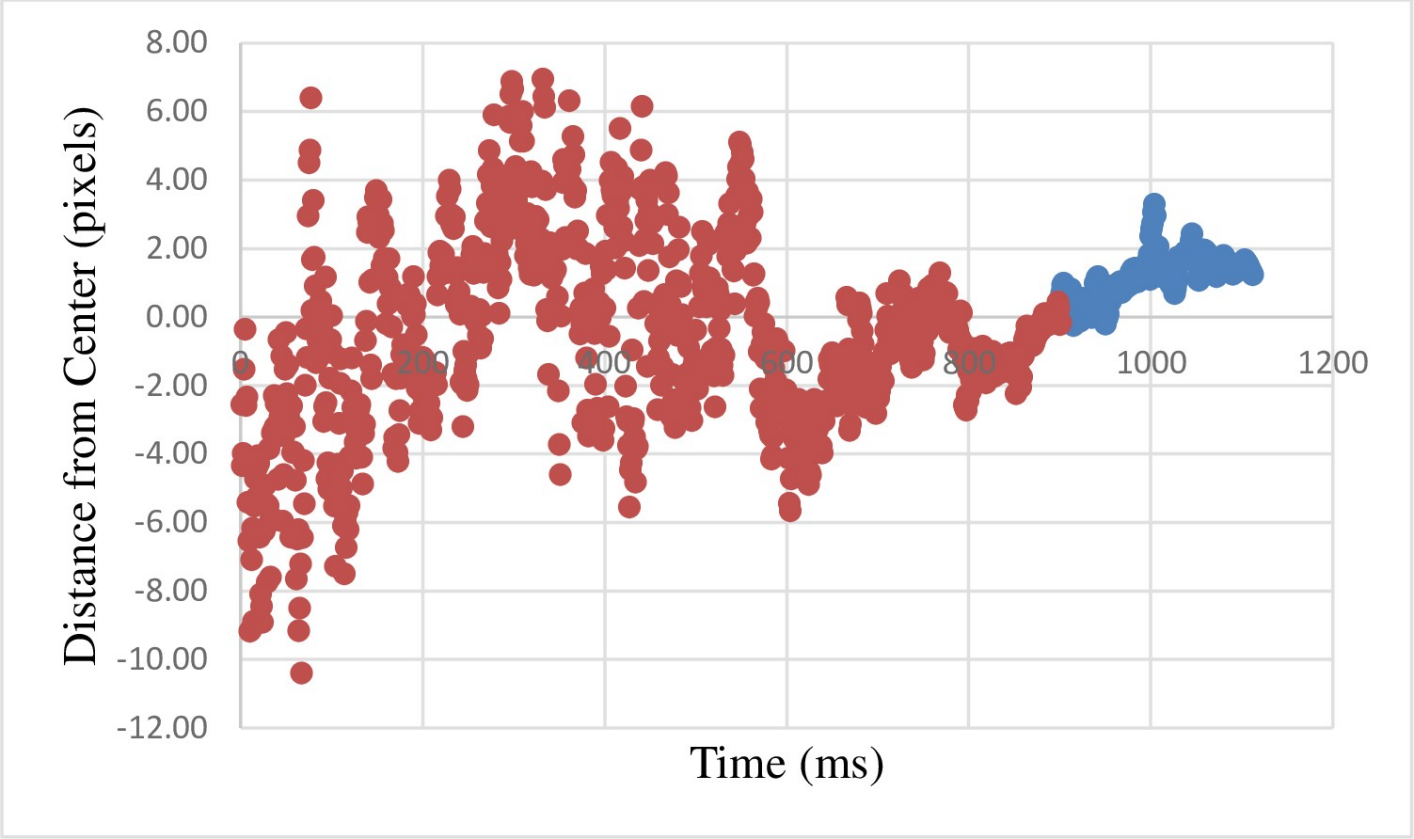
The graph describes, for each time point, the mean distance of the gaze from the center of the screen (which is also the final point of masked probe). Each point is the average across all participants and all trials. Red marks the average gaze during the masked probe presentation. Blue marks the average gaze during the delay period–between the offset of the masked probe and the onset of the conscious target. 1-pixel equals 0.024-degree in the visual field.

For the participants who were classified as nonconscious we found a positive correlation between the time when the probe disappeared and the distance of the gaze from the center (r(209) = 0.65, p < .001). This correlation shows that the gaze is moving away from the fixation in the direction inferred from the motion. See Fig 9 and S3 Appendix (shows a version of Fig 9 with error bars). For the correlations in the different directions see S5 Appendix. Importantly, in five directions the results turned out positive and significant as well. In one direction it was negative and marginally significant (see S5 Appendix).

#### Further analyses

The results suggest that 600 ms after the beginning of the motion, the eyes track the masked probe. This is indicated by positive correlation between the distance of the gaze from the center, and time between 600 ms and the rest of the masked presentation (r(299) = 0.57, p < .001). As the choice of the 600 ms time was obtained only after obtaining this result it is of most importance to replicate it. However, if replicated, it demonstrates the importance of eye-tracking in masked methodology, as it seems to be, at least in this case, more sensitive than behavioral variables.

The Behavioral results did not support our hypothesis, as participants were not significantly faster in the future condition (M = 0.55, seconds, SD = 0.12) than in the control condition (M = 0.55 seconds, SD = 0.11), (t(17) = 0.142, p = .89). Greenwald et al.’s (1995) regression did not reveal a significant intercept (b0 = 0.99, t(28) = 0.21, p = .83). As the behavioral results from the non-conscious participants were not significant, nor was the intercept, we will not report here the results of the Bayesian correlation and awareness test reliability. The graphs and scatter plot for this experiment displayed in S4 Appendix.

The slope did not turn out significant either (b = -5.3, t(28) = 0.206, p = 0.84). In the supraliminal training phase participants were not significantly faster in the future condition (M = 0.53 seconds, SD = 0.08) than in the control condition (M = 0.54, seconds, SD = 0.1) (t(17) = 1.03, p = .316, d = 0.24).

To summarize, while the behavioral effect was not replicated, a reliable eye-tracking effect was measured. As described, in order to take eye-tracking measurement during CFS we are using a new technology, namely, an alternating screen which is originally used for 3D movies. However, in contrast to 3D movies the visual stimuli to each of the eyes are very different. This causes a residue of the mask on the screen while the masked probe is presenting. This noise may load attention and hinder the behavioral effect.

## Experiment 6

In an attempt to replicate the behavioral and the eye-tracking effects while dealing with the residue of the mask we replicated the previous experiment with increased maximal contrast of the probe.

### Method

#### Participants

Given the difficulty to estimate the number of participants that will be included in the final experiment we decided to stop after 35 participants that are masked and have accuracy performance above 90%. Seventy-seven (thirty-four males; M = 25.43 years, SD = 3.56) with intact vision (without glasses or contact lenses) participated in the experiment. They received either 10 NIS (~$3) or course credit. Prior to their participation all participants gave an informed consent by signing a written consent form.

#### Procedure

The procedure was identical to that of the previous experiment, except that the maximum contrast was increased to 85%.

### Results & discussion

#### Data preparation

We followed the preprocessing procedures from previous experiment. These include exclusions of participants and trials: Forty participants whose scores in the awareness test (phase 3) deviated from chance were classified as conscious, and the rest were classified as nonconscious. One participant with faulty eye-tracking was removed for the analysis. The participant with faulty recording was eligible for behavioral analysis but was excluded due to the loss of eye-tracking data. Two participants whose accuracy was below 90% in the masked presentation (phase 2) were excluded from the analyses. After the exclusion of participants, erroneous trials (i.e., trials in which participants made mistakes in the classification task) were excluded from the analyses (3.5%), as were trials with reaction times (RTs) longer than 5 seconds or shorter than 0.2 seconds (less than 0.5%), and likewise trials with RTs that deviated more than 3 standard deviations from each participant’s mean (1.5%).

#### Awareness

The nonconscious classified group’s mean accuracy in the awareness test (M = .5, SD = 0.07) did not deviate from chance (t(33) = 0.32, p = .75).

#### Main results

**Fig 10:**
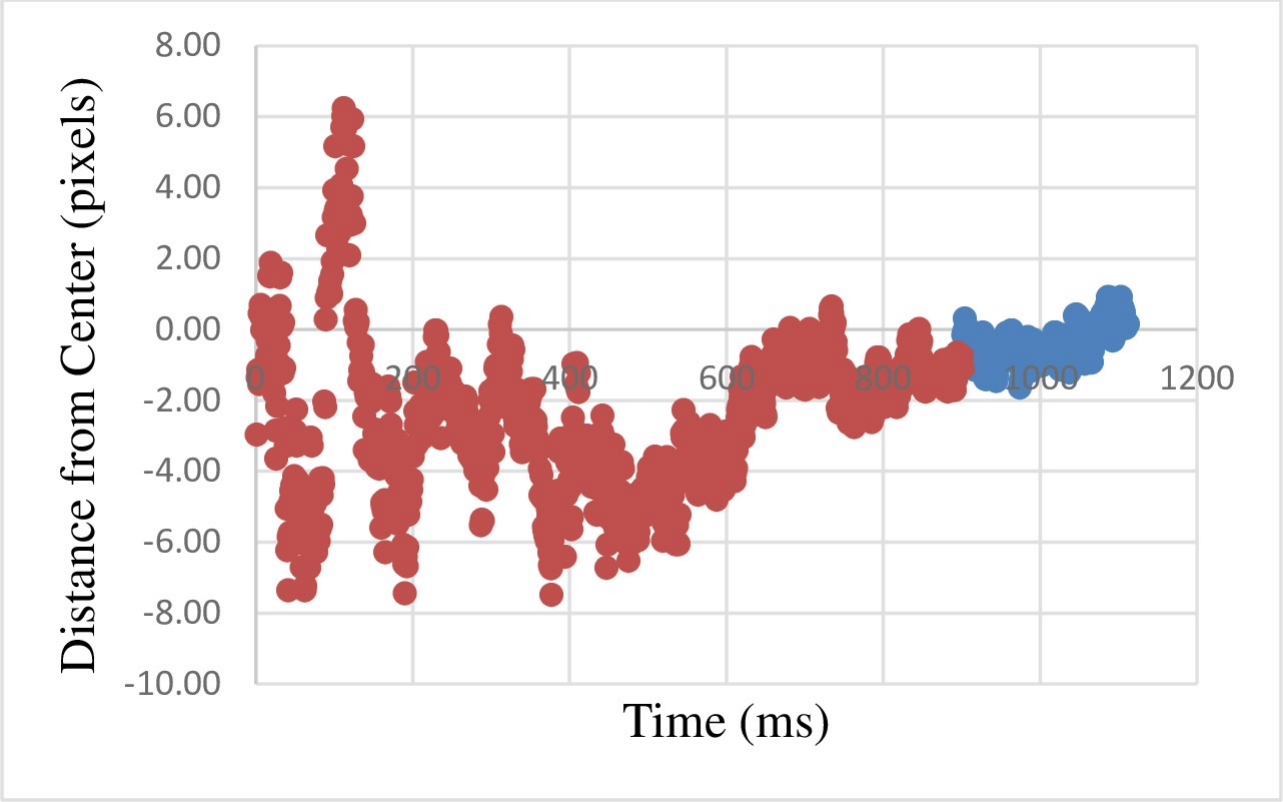
The graph describes, for each time point, the mean distance of the gaze from the center of the screen (which is also the final point of the masked probe). Each point is the average across all participants and all trials. Red marks the average gaze during the masked probe presentation. Blue marks the average gaze during the delay period–between the offset of the masked probe and the onset of the conscious target. 1-pixel equals 0.024-degree in the visual field.

As expected for the participants who were classified as nonconscious we found a positive correlation between the time when the probe disappeared and the distance of the gaze from the center (r(209) = 0.57, p < .001). This correlation shows that the gaze is moving away from the fixation in the direction inferred from the motion. See Fig 10 and S3 Appendix. For the correlations in the different directions see S5 Appendix. Importantly, in five directions the results turned out positive and significant as well. In three directions the correlation turned out negative and significant (see S5 Appendix). The negative correlations suggest that there might be another effect taking place in addition to gaze motion in the direction inferred motion. For example, it is possible that for some directions (some) participants are moving their eyes back to the fixation point during the delay. This will generate a negative correlation as, in this case, the distance of the gaze from fixation point will get smaller in time. Importantly, as the experiments are fully counterbalanced (and symmetric in terms of the directions), the aggregated results support the claim of gaze-motion in the direction of the inferred motion.

#### Further analyses

We replicated the correlation between the time and probe distance in the interval that starts 600 ms after the onset of the prime until the end of the presentation (r(299) = 0.24, p<0.001). This supports the hypothesis of active prediction of masked stimuli as well the fact that in some cases eye-tracking indicates cognitive processes that reaction times do not necessarily indicate.

The behavioral results did not support our hypothesis, as participants were not significantly faster in the future condition (M = 0.61 seconds, SD = 0.08) than in the control condition (M = 0.62 seconds, SD = 0.07), (t(33) = 0.88, p = 0.39). Greenwald et al.’s (1995) regression did not reveal a significant intercept (b0 = 3.01, t(69) = 0.9, p = 0.34). As the behavioral results from the non-conscious participants were not significant, nor was the intercept, we will not report here the results of the Bayesian correlation and awareness test reliability. The scatter plot for this experiment displayed in S2 Appendix.

The slope did not turn significant either (b = 14.47, t(69) = 0.99, p = 0.33). In the supraliminal training phase participants were significantly faster in the future condition (M = 0.59 seconds, SD = 0.09) than in the control condition (M = 0.62 seconds, SD = 0.12) (t(33) = 2.91, p = .006, d = 0.5).

One possible reason for the failure of the behavioral effect in Experiments 5 and 6 has to do with the distance between the participants and the screen, which was doubled in Experiments 5 and 6 (60 cm vs. 30 cm) in order to enable good eye tracking signal. This change results in smaller visual angle of the stimuli and stimuli movement, and shorter visual angle between the probe and the target, which may yield smaller effects. Our results may then suggest that in some cases eye-tracking is a more sensitive measure of non-conscious processes and may be used to shed light on the mechanism of prediction that are not accessed by merely measuring reaction times.

### Aggregated analyses

A conservative test for non-conscious processing is taking a threshold of 0.5 (chance level) for classifying participants according to the awareness test. While this threshold gives a high degree of confidence that the effect measured (if significant) is non-conscious, it has a big caveat of excluding a big portion of the participants. Thus, we used this threshold on the aggregated data from Experiments 1–6 (N = 71). We conducted a mixed design with the experiment as a between-subject condition and experimental condition (control condition in comparison to future condition). The within-subject condition turned out significant (F(1,65) = 26.722, p < .001), giving strong evidence that the effect of active prediction under masked condition. In addition, the interaction turned out significant as well (F(1,5) = 6.823, p < .001), suggesting the differences between Experiments 5 and 6 and Experiments 1–4 attenuated the behavioral effect. Experiments 5 and 6 have a different setup than Experiments 1–4 as instead of mirror stereoscope they make use of shutter-glasses. In addition, from a perceptual perspective they also differ in the mask they use. The masks in Experiments 5 and 6 consist of filled circles. It is possible that this attribute may capture awareness more than the empty ones in Experiment 1 to 4.

We also conducted Greenwald regression for the collapsed data from Experiments 1–6, for participants whose accuracy in the conscious target classification was above 90% in the masked presentation part (N = 258). The intercept turned out significant (t(257) = 5.725, p < .001).

## General discussion

Six experiments demonstrated that humans generate active predictions from masked dynamic events. The first two experiments examined direction-based predictions and the third examined speed-based ones. The fourth experiment required a more complex understanding that involved collision and deflection, and here, too, participants were able to integrate the masked information and to actively generate predictions. The fifth and sixth experiments provided physiological data–in the form of eye movements–that supported the idea of prediction induced by masked dynamical stimuli.

We believe that the different analyses we report support the hypothesis that the stimuli were rendered subliminal, and that partial awareness cannot account for our results. Thus, one can conclude that humans can actively generate predictions even for events that are entirely outside of conscious awareness. However, we acknowledge that analyses that are based on objective awareness tests cannot ensure beyond all doubts that some masked trials were not rendered supraliminal. In this case, if these were treated as subliminal, it would be possible for them to contribute or even drive the measured effects. Let us first discuss the nature and implications of our findings, and then turn to an elaborate discussion of the issue of subliminality.

The stimuli in our experiments consist of a probe moving in a linear line. As the predictions are based on the movement of a probe, they inherently testify to nonconscious temporal-spatial integration. The debate regarding the scope of nonconscious integration is vibrant and new findings and arguments are consistently being introduced [138]. One school of thought suggests that information integration is necessarily conscious (it constitutes consciousness), thereby implying that non-conscious integration is impossible [139–141]. However, recent studies and reviews show that in different domains information is being integrated non-consciously [50, 142, 143] (for a short review see [138]). Our results add support to the idea that humans’ nonconscious processes can integrate information along space and time.

Another discussion our experiments are relevant to is the issue of inducing endogenous attention using subliminal stimuli. Gayet, Van der Stigchel and Paffen [68] showed that the subliminal arrow symbol can cue a spatial location congruent with the direction the arrow is pointing at. However, they also showed that this facilitation is dependent on intermixing supraliminal predictive cues of the same type. As acknowledged, it is possible that some participants that are classified as nonconscious are in fact conscious of some masked trials. It is likely that these trials will be mostly congruent (as in most experiments most trials are congruent, and thus predictive). Thus, it is possible, that these few conscious trials are satisfying the condition that Gayet, Van der Stigchel and Paffen [68] outlined for endogenous subliminal attention. However, it is also possible that the stimuli we used (i.e motion) can facilitate this shift of attention without the need of intermixing conscious trials. Support for this claim we can draw from the work of Rahnev, Huang and Lau [67] demonstrating endogenous attention from nonconscious motion (though not facilitation of shift in spatial attention).

The predictions examined here are based on a specific type of information–movement–and they project only hundreds of milliseconds into one’s future. Humans, however, possess a unique ability to predict their distant future and use these predictions to guide present behavior [18]. Previous research has shown that one need not be aware of the fact that she makes these predictions, nor of their content [9, 144]. Yet, the predominant use of conscious stimuli in previous research [10, 145, 146] may suggest that the events on which these predictions are made must be consciously experienced. The present results suggest that this might not be the case. Rather, we can generate these kinds of predictions even for events that we do not consciously notice in our lives. This intriguing question is left for future investigations.

Our research replicates the finding of Hsieh and Colas [62] regarding activation of past location (Experiment 1). However, there is an apparent discrepancy as their experiment did not facilitate active predictions regarding the future location. This is potentially related to the type of motion used. In their research they did not use continuous moving probes, but rather stationary probes that were displaced in space every 600 milliseconds, potentially creating *apparent motion*. They used this type of motion to test if awareness is necessary for extracting patterns in working memory. Our research focuses on predictions and not necessarily on predictions that require pattern extraction from visual working memory. In addition, Recent research suggests that apparent motion may decrease under load [147]. It may well be the case, then, that apparent motion is also more fragile under subliminal conditions, thus explaining the null results reported by Hsieh and Colas [62]. Further research is needed in order to examine this issue.

There are intimate connections between current research and the literature on the (conscious) representational momentum [13]. The literature on Representational Momentum covers predictions based on motion [114] and also includes deflection from barriers predictions [102, 148]. In these experiments participants misremember the last location of a moving object, “moving” it to a location that it did not get to yet, that is, into its future. However, the movement used in this literature is *always supraliminal*, and thus our experiments shed light on the necessity of consciousness in this literature.

Yet, it is interesting to also note the similarities between our findings and those reported for the conscious momentum effect. The facilitation along the future route (same-direction conditions) in comparison to the control condition (in Experiments 1, 2, 5 and 6) is common to both [95, 149, 150]. The sensitivity to the velocity of the moving probe and its effect on prediction (Experiment 3) has also been documented in both [151, 152].

It has been established that some predictions induced by motion require semantic knowledge, planning, working memory, and attentional control [95, 150, 153–155]. Our methodology opens the door to exploring these and other high-level cognitive functions non-consciously using continuous motion and stimuli that is motion based.

In order to support the claim that the masked presentation induced nonconscious processes we used an awareness-test phase. We use the performance in awareness-test phase in two complementary analyses. The first analysis uses that score to classify nonconscious participants. This method is criticized by Shanks [74] as an invalid one. The criticism is formulated in terms of regression to the mean, the claim is that the actual score of the classified groups is closer to the population mean which means that the classified group is not in fact nonconscious. A crucial point, that is often over-looked, is that this is a problem if and only if partial awareness can induce the behavioral findings. Partial awareness is awareness of features of the masked stimulus as it is being masked. If being partially aware (as many participants clearly do), does not contribute to the measured effect, then, even if the actual awareness score is higher, it will not immediately nullify the results. Therefore, in Experiments 1, 3 and 4 where Bayes factor suggests strong evidence that the correlation between the behavior effect and awareness score is not positive this criticism is not valid.

This leads to the second analysis we implemented. This analysis is inspired by Greenwald et al [90]. It is complementary to the classification-based analysis as it does not rely on classifying participants thus avoid the issue raised by Shanks [74]. The method relays on testing the significance of the intercept in a linear regression where the effect is predicted from the awareness score. It was noted that due to error measurement, in cases where the slope of the linear regression is positive the intercept will be overestimated, but if negative, it will be underestimated. This means that if the slope of Experiment 1 had reached significance it would have meant that the non-conscious effect is actually higher than the one estimated by the intercept. In addition, and as stated before, in Experiments 1, 3 and 4 Bayes factor indicated strong evidence that the correlation between the behavior effect and awareness score is not positive, suggesting that the correlation between the awareness score and effect size is not likely to bias the intercept. We acknowledge that even high Bayes factors cannot definitively exclude the possibility that there is in fact a positive correlation between awareness score and the effect. Thus, one may still have doubts whether the entire or some of the processes required are indeed nonconscious.

A direct approach to the problem of overestimation of nonconscious effects of Greenwald’s regression due to error-in-measurement is described in Goldstein, Sklar and Siegelman [156]. This implements the generative Bayesian modeling approach [157] which already was proved useful for calculating correlation in the presence of measurement error [158]. This was generalized to the analysis of linear regression allowing for following Greenwald’s regression analysis while controlling of the influence of measurement error.

Another version of the criticism, is that because the awareness is tested separately from behavior effect, it is possible that the measured effect is a result of a few conscious trials in the subliminal part of the experiment [41]. We acknowledge, that while unlikely, it is still possible that participants that were classified as nonconscious were in fact aware of (a small portion of) trials. In addition, we are aware of the controversy around using objective tests for establishing subliminality (and especially ones that use threshold performance).

While we believe that it is more likely that participants will be partially aware (and not fully aware), and that Greenwald analysis should suffice examine the effect, we offer a new type of analysis that directly deals with this possible criticism. In this analysis we simulate the effect of conscious trials (by sampling them from the supraliminal training phase) and mix them with the reaction times measured in the masked part after shuffling the condition labels (to match the hypothesis that they do not contribute to a systematic effect). In Experiments 1–4 where reaction times indicated non-conscious effect, the simulations strongly suggest that the measured effects in the masked phases are not likely to be a result of a few conscious trials.

In addition, in Experiments 1–3 non-conscious effects were found but conscious effects were not recorded. While in Experiment 6 a conscious effect was found but not a non-conscious one. This cast doubt on the relevance of the criticism that conscious trials mixed in the subliminal part are yielding the effect.

Another approach that theoretically could be used to avoid the concern of conscious trials mixing in the subliminal part implements trial-by-trial awareness measurement. In this methodology after each trial in the masked phase participants are asked to report whether, or to what extent they were conscious of the subliminal stimulus [62, 63, 82, 121, 159]. The advantage of this methodology is that it does not involve inferring the awareness level in the masked phase from a different phase. Thus, it directly assesses the awareness to the relevant trials. While this is a legitimate approach it is fundamentally exposed to the same type of criticism, as it is exposed to measurement errors as any other behavioral measure is.

The root of the criticism on the methodology we chose is error in measurement (or measurement error). It lies at the center of Shank’s criticism; the overestimation claims about Greenwald analysis and also the claim that few conscious trials could mix in the masked phase. Error measurement is part of every psychological measurement, including trial-by-trial awareness measurement. Participants can make mistakes in this classification the same as in any other task. The advantage of using awareness test phase is that it allows to estimate the error measurement by calculating the reliability of awareness. In addition, using the phase-awareness-test allows us to explore the influence of error-in-measurement on the results. For example, in the case of Greenwald analysis, error measurement causes an overestimation in case of positive slope but underestimation in case of negative slope. It is less clear how to count for the influence of error in measurement in trial-by-trial awareness measurement, thus we hold that it is not necessarily a better approach than the one taken for this paper.

The methodology of awareness test phase allows to estimate the reliability of the awareness testing. Indeed, the reliability of the awareness test in our experiments are relatively high (higher than 0.8). However, this comes at a cost of having a large number of participants with scores indicating some level of partial awareness. This is of course not an issue for Greenwald analysis, but it poses a problem when classifying nonconscious participants. The main problem with an “ideal” mask where all participants perform at chance level is that it will be indistinguishable from a situation of participants who are simply not performing the task.

To summarize the methodological aspect of this discussion we would like to acknowledge that the research of non-conscious is currently intertwined with methodological discussions as to the proper ways of manipulating and testing awareness. In this paper we offer a new simulation-based analysis as well as nuanced approach to the different types of analyses and criticisms that are already documented in the literature. We hope that future research will allow to directly estimate non-conscious effects while counting the reliability and error in measurement issues. However, we believe that the empirical research should not halt until all the methodological issues are solved and that the more data are available the better the methodologies will become.

## Supporting information

S1 Appendix Online materials. (DOCX)

S2 Appendix Repeated measure analysis with Angle factor. (DOCX)

S3 Appendix Eye tracking results with standard errors. (DOCX)

S4 Appendix Scatter plots for Experiments 5 and 6. (DOCX)

S5 Appendix Correlation between index and gaze distance for each direction. (DOCX)
