# Supplementary material for: Predictions from masked motion with and without obstacles
Source: PLoS One. 2020 Nov 6;15(11):e0239839. doi: 10.1371/journal.pone.0239839 (PMC7647069; doi:10.1371/journal.pone.0239839)
Supplement: S1 Appendix — (DOCX) [file pone.0239839.s001.docx]

S1 Appendix – Online materials

This ReadMe file describes the datasets and procedures regarding the paper

Predictions from masked motion with and without obstacles.

It provides instructions how to reproduce the results in the paper. The shared repository consists of two folders:

Behavior and ET.

Behavior

The Behavior folder consists of the dataset and script that yield the behavioral results of Experiments 1-6. Experiments 5&6 main purpose is the Eye-Tracking (ET) results, however the same procedures in regard to the behavioral results were implemented. The folder consists of 3 files:

1. Behavior_Data.xlsx

2. Analysis_Behavior_Data.sps

3.Behavior_Data.txt

Behavior_Data.xlsx - consists of the data per each trial per each participant per each experiment.

Behavior_Data.txt - consist of the commands in Analysis_Behavior_Data.sps for those who do not have access to SPSS.

How to reproduce the results:

Analysis_Behavior_Data.sps - is an SPSS syntax (SPSS 25 for MAC). In order to run the script (using SPSS) one should change line 8 and give the proper reference to Behavior_Data.xlsx.

Eye-Tracking

The ET folder consists of the datasets and scripts that yield the eye-tracking results of Experiments 5&6.

How to reproduce the results.

1. After downloading ET folder (with all the subfolders and scripts).

2. Run Main.m with matlab (2017a)

3. This will generate 'exp_5_6_prime_dely.txt’

4. This file should be converted to an SPSS datafile (say file) (using the titles as names of variables). The name should be Experiments_5_6.sav

5 In Exp5_6_Prime_Dely.sps In line 4 one should change and give the proper reference to Experiments_5_6.sav

6. Run the SPSS script file Exp5_6_Prime_Dely.sps
