# Supplementary material for: Predictions from masked motion with and without obstacles
Source: PLoS One. 2020 Nov 6;15(11):e0239839. doi: 10.1371/journal.pone.0239839 (PMC7647069; doi:10.1371/journal.pone.0239839)
Supplement: S2 Appendix — (DOCX) [file pone.0239839.s002.docx]

S2 Appendix – Repeated measure analysis with Angle factor

| Angle (PI) | Experiment 1 (N=18) | | | |
| --- | --- | --- | --- | --- |
|  | Control | | Future | |
|  | Mean (sec) | SD | Mean (sec) | SD |
| 0 | 0.6202 | 0.16013 | 0.6478 | 0.24945 |
| 0.25 | 0.7231 | 0.24601 | 0.6523 | 0.20716 |
| 0.5 | 0.6126 | 0.11543 | 0.6358 | 0.20503 |
| 0.75 | 0.673 | 0.17617 | 0.6135 | 0.15057 |
| 1 | 0.6646 | 0.20455 | 0.6223 | 0.2113 |
| 1.25 | 0.6635 | 0.18033 | 0.6058 | 0.11106 |
| 1.5 | 0.6962 | 0.21207 | 0.6226 | 0.18385 |
| 1.75  Table 1 | 0.7126 | 0.26311 | 0.647 | 0.17532 |

In a repeated measure analysis we introduced the Angle and Condition as within-subject factors the Condition factor turned out significant (F(1,17)=12.73, p=.002), the Angle factor turned out significant (F(7,17)=2.99, p=.006), the interaction turned out significant as well (F(7,17)=2.205, p=.039).

| Angle (PI) | Experiment 2 (N=11) | | | |
| --- | --- | --- | --- | --- |
|  | Control | | Future | |
|  | Mean (sec) | SD | Mean (sec) | SD |
| 0 | 0.5872 | 0.06956 | 0.566 | 0.0731 |
| 0.25 | 0.6122 | 0.08315 | 0.5917 | 0.07189 |
| 0.5 | 0.5937 | 0.0702 | 0.5789 | 0.10758 |
| 0.75 | 0.6292 | 0.07364 | 0.5639 | 0.08813 |
| 1 | 0.6005 | 0.10767 | 0.5412 | 0.08113 |
| 1.25 | 0.604 | 0.05892 | 0.5635 |  |
| 1.5 | 0.6133 | 0.09104 | 0.6073 | 0.11629 |
| 1.75  Table 2 | 0.5967 | 0.0889 | 0.582 | 0.05103 |

In a repeated measure analysis we introduced the Angle and Condition as within-subject factors the Condition factor turned out significant (F(1,10)=11.84, p=.006), the Angle factor did not turned out significant (F(7,10)=1.67, p=.13), the interaction did not turned out significant as well (F(7,10)=1.08, p=.385).

| Angle (PI) | Experiment 3 (N=28) | | | |
| --- | --- | --- | --- | --- |
|  | Control | | Future | |
|  | Mean (sec) | SD | Mean (sec) | SD |
| 0 | 0.6009 | 0.11628 | 0.5763 | 0.11233 |
| 0.25 | 0.6182 | 0.15047 | 0.5918 | 0.11706 |
| 0.5 | 0.5685 | 0.11619 | 0.5636 | 0.0832 |
| 0.75 | 0.5764 | 0.1085 | 0.5924 | 0.11075 |
| 1 | 0.5818 | 0.14789 | 0.5828 | 0.1153 |
| 1.25 | 0.6111 | 0.16843 | 0.5847 | 0.11523 |
| 1.5 | 0.6123 | 0.1865 | 0.5845 | 0.11672 |
| 1.75  Table 3 | 0.6028 | 0.12416 | 0.5852 | 0.10273 |

In a repeated measure analysis we introduced the Angle and Condition as within-subject factors the Condition factor turned out significant (F(1,27)=5.515, p=.026), the Angle factor did not turned out significant (F(7,27)=1.48, p=.18), the interaction did not turned out significant as well (F(7,27)=0.626, p=.75).

| Angle (PI) | Experiment 5 (N=18) | | | |
| --- | --- | --- | --- | --- |
|  | Control | | Future | |
|  | Mean (sec) | SD | Mean (sec) | SD |
| 0 | 0.5598 | 0.15733 | 0.557 | 0.09937 |
| 0.25 | 0.564 | 0.13467 | 0.5578 | 0.11118 |
| 0.5 | 0.5566 | 0.11211 | 0.5815 | 0.2186 |
| 0.75 | 0.5796 | 0.15978 | 0.5554 | 0.10995 |
| 1 | 0.5559 | 0.12347 | 0.5387 | 0.09916 |
| 1.25 | 0.5614 | 0.16282 | 0.5474 | 0.15463 |
| 1.5 | 0.541 | 0.10736 | 0.5684 | 0.14998 |
| 1.75  Table 4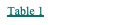 | 0.5379 | 0.08801 | 0.5564 | 0.13031 |

In a repeated measure analysis we introduced the Angle and Condition as within-subject factors the Condition factor did not turned out significant (F(1,17)<1), the Angle factor did not turned out significant (F(7,17)<1), the interaction did not turned out significant as well (F(7,17)<1).

| Angle (PI) | Experiment 6 (N=34) | | | |
| --- | --- | --- | --- | --- |
|  | Control | | Future | |
|  | Mean (sec) | SD | Mean (sec) | SD |
| 0 | 0.6022 | 0.09282 | 0.6143 | 0.08566 |
| 0.25 | 0.6112 | 0.09776 | 0.6184 | 0.07475 |
| 0.5 | 0.6313 | 0.0904 | 0.619 | 0.0902 |
| 0.75 | 0.6178 | 0.08566 | 0.6039 | 0.07146 |
| 1 | 0.6351 | 0.08341 | 0.6105 | 0.08979 |
| 1.25 | 0.6165 | 0.09241 | 0.5954 | 0.07321 |
| 1.5 | 0.6233 | 0.09565 | 0.623 | 0.08991 |
| 1.75 | 0.5978 | 0.10091 | 0.6277 | 0.09552 |

Table 5
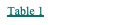


In a repeated measure analysis we introduced the Angle and Condition as within-subject factors the Condition factor turned did not turned out significant (F(1,33)<1), the Angle factor did not turned out significant (F(7,33)=1.47, p=0.179), However, the interaction did turn out significant (F(7,33)=2.27, p=.03).
