# Supplementary material for: Predictions from masked motion with and without obstacles
Source: PLoS One. 2020 Nov 6;15(11):e0239839. doi: 10.1371/journal.pone.0239839 (PMC7647069; doi:10.1371/journal.pone.0239839)
Supplement: S3 Appendix — (DOCX) [file pone.0239839.s003.docx]

S3 Appendix – Eye tracking results with Standard Errors

Time (ms)

Distance from Center (pixels)

Experiment 6


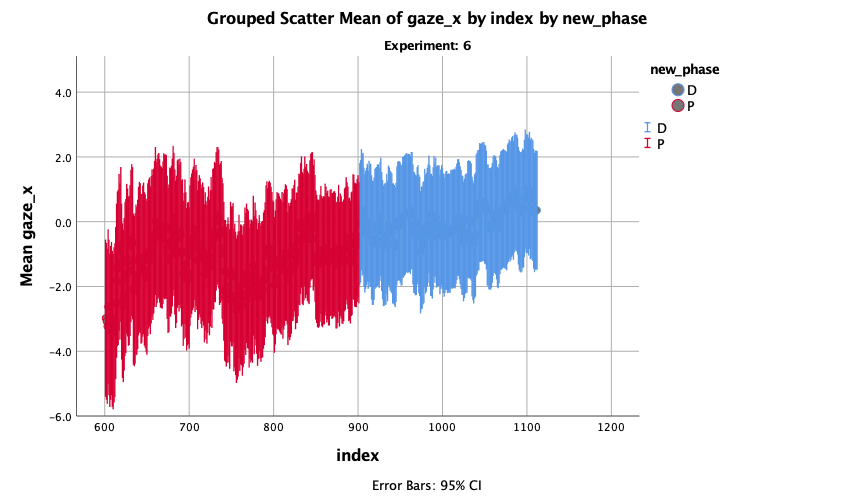

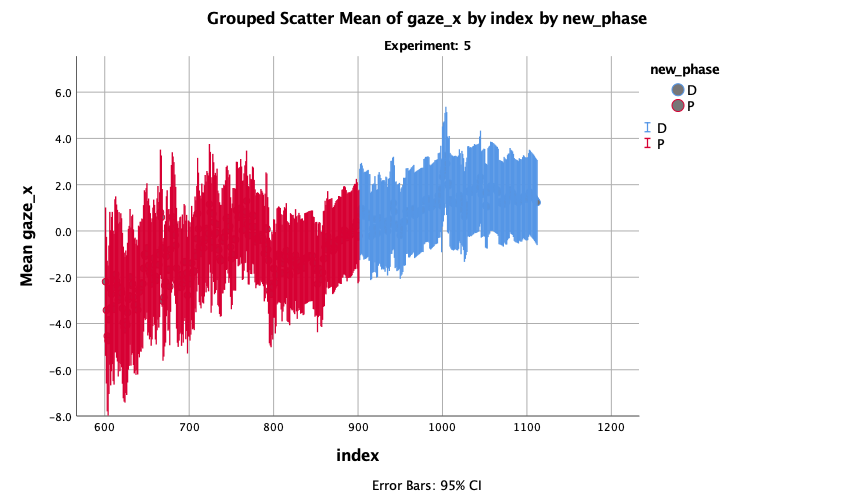


Time (ms)

Distance from Center (pixels)

Experiment 5

The graph describes, for each time point, the mean with the standard error of the distance of the gaze from the center of the screen (which is also the final point of subliminal probe). Each point is the average across all participants and all trials. Red marks the average gaze during the subliminal probe presentation. Blue marks the average gaze during the delay period – between the offset of the subliminal probe and the onset of the conscious target.

1-pixel equal 0.024-degree in the visual field
