# Supplementary material for: Predictions from masked motion with and without obstacles
Source: PLoS One. 2020 Nov 6;15(11):e0239839. doi: 10.1371/journal.pone.0239839 (PMC7647069; doi:10.1371/journal.pone.0239839)
Supplement: S4 Appendix — (DOCX) [file pone.0239839.s004.docx]

S4 Appendix – Scatter plots for Experiments 5 and 6

Experiment 5
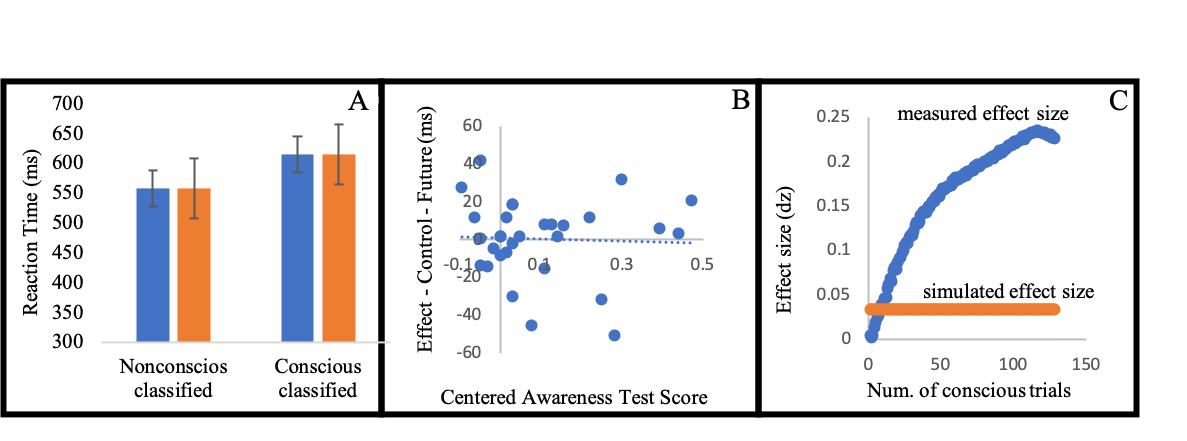


For both level of awareness (panel A) the difference between future and control did not yield a significant difference (t<1).

Experiment 6


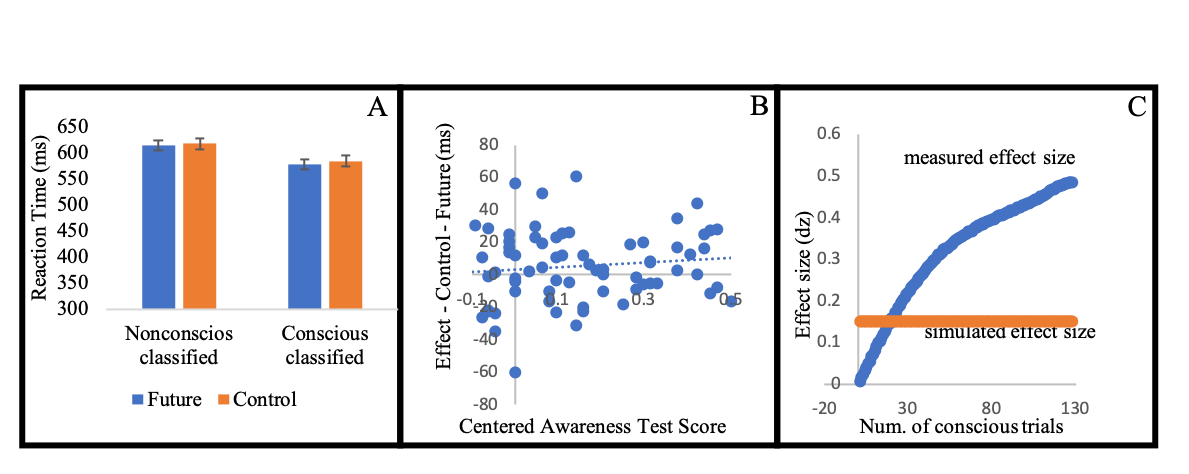


Participants at the conscious classified condition participants were faster in the future condition than control condition (t(36)=2.055, p=0.047).
