# Supplementary material for: Predictions from masked motion with and without obstacles
Source: PLoS One. 2020 Nov 6;15(11):e0239839. doi: 10.1371/journal.pone.0239839 (PMC7647069; doi:10.1371/journal.pone.0239839)
Supplement: S5 Appendix — (DOCX) [file pone.0239839.s005.docx]

S5 Appendix – Correlation between index and gaze distance for each direction

| Experiment 5 | | |
| --- | --- | --- |
| Angle (PI) | r | p |
| 0.25 | 0.213 | 0.002 |
| 0.5 | 0.06 | 0.387 |
| 0.75 | 0.277 | <0.001 |
| 1 | 0.209 | 0.002 |
| 1.25 | 0.428 | <0.001 |
| 1.5 | -0.115 | 0.096 |
| 1.75 | 0.548 | <0.001 |
| 2 | -0.132 | 0.056 |

| Experiment 6 | | |
| --- | --- | --- |
| Angle (PI) | r | p |
| 0.25 | -0.241 | <0.001 |
| 0.5 | 0.736 | <0.001 |
| 0.75 | 0.755 | <0.001 |
| 1 | 0.932 | <0.001 |
| 1.25 | -0.867 | <0.001 |
| 1.5 | -0.335 | <0.001 |
| 1.75 | 0.14 | 0.043 |
| 2 | 0.826 | <0.001 |
